# Programmable and Reversible 3D‐to‐3D Shape Transformation: Hierarchical Multimodal Morphing Based on Liquid Crystal Elastomers

**DOI:** 10.1002/advs.202507922

**Published:** 2025-07-28

**Authors:** Jiayu Tian, Chuanqian Shi, Guohua Nie, Chenzhe Li, Ying Zhao

**Affiliations:** ^1^ School of Aerospace Engineering and Applied Mechanics Tongji University 100 Zhangwu Road Shanghai 200092 China; ^2^ Center for Mechanics Plus under Extreme Environments School of Mechanical Engineering & Mechanics Ningbo University 100 Fenghua Road Ningbo 315211 China

**Keywords:** 3D‐to‐3D shape morphing, hierarchical multimodal transformations, LCE‐Ela bilayer, mismatch strain driven, programmable and reversible

## Abstract

Achieving programmable morphing in 3D‐to‐3D shapes of soft actuators based on liquid crystal elastomer (LCE), particularly those with multimodal transformations and non‐zero Gaussian curvature, remains a significant challenge. Here, a facile strategy is presented to create 3D LCE‐elastomer (LCE‐Ela) bilayer structures capable of customizable and programmable 3D‐to‐3D shape transformations, generating reversible and multimodal morphologies with nonzero Gaussian curvature. By combining two types of mismatch strains—pre‐stretch and thermal strains—in LCE‐Ela bilayer, the approach enables hierarchical multimodal transformations: starting from a 2D initial configuration, programmable transformations enable the formation of complex 3D structures, which can subsequently transition into other 3D shapes following predefined programs, with each step of the hierarchical process allowing multimodal deformations to generate diverse structural morphologies. Experimental and computational demonstrations include over 30 diverse 3D LCE‐Ela configurations, the majority of which exhibit nonzero Gaussian curvature. Moreover, biomimetic LCE‐Ela structures—including a chameleon, butterfly, spider, and leaf—demonstrate vivid deformation and discoloration, showcasing their potential for applications such as information encryption, camouflage, and adaptive devices. This work provides a facile approach to generate customizable 3D‐to‐3D transformations with complex geometries, broadening the application scope of LCE‐based technologies in 3D soft actuators.

## Graphical abstract

Hierarchical multimodal 3D‐to‐3D shape morphing is achieved in liquid crystal elastomer‐elastomer bilayers via programmable mismatch strains. Combining pre‐stretch and thermal actuation enables reversible transformations with non‐zero Gaussian curvature, empowering over 30 complex configurations for biomimetic camouflage, adaptive robotics, and dynamic information encryption.

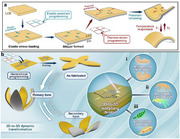

## 1 Introduction

Natural species demonstrate remarkable abilities to strategically transform their shapes for adaptive and complex purposes^[^ ^1^, ^2^ ^]^ such as the self‐burial drilling motion of wheat awns for seed dispersal^[^ ^3^ ^]^ and the trap closing in the carnivorous Dionaea muscipula for capturing prey.^[^ ^4^ ^]^ Inspired by the adaptive shape transformations found in natural species, scientists have recently developed a wide range of shape‐shifting structures that respond to specific stimuli, including mechanical forces,^[^ ^5^ ^]^ magnetic fields,^[^ ^6^, ^7^ ^]^ heat,^[^ ^8^, ^9^ ^]^ light,^[^ ^10^, ^11^ ^]^ and electricity. Through structural design and material distribution, programmable transformations from an initial flat shape into a predetermined 3D form can be achieved. These programmable shape‐shifting structures have significant potential in applications across diverse fields, such as soft robotics,^[^ ^12^, ^13^ ^]^ bioengineering,^[^ ^14^, ^15^ ^]^ and flexible devices.^[^ ^5^, ^16^ ^]^ Typically, the fabrication of such structures relies on stimuli‐responsive materials, including hydrogels,^[^ ^17^, ^18^ ^]^ shape memory polymers,^[^ ^19^, ^20^ ^]^ magnetically responsive composites,^[^ ^6^, ^7^ ^]^ and liquid crystal elastomers (LCEs).^[^ ^21^, ^22^ ^]^

Among these structures, shape‐shifting systems based on LCEs have garnered significant attention due to their soft elasticity, stable and repeatable performance, and the ability to undergo large, reversible shape changes in response to external stimuli, such as heat,^[^ ^8^, ^9^ ^]^ electric heat,^[^ ^23^ ^]^ and light.^[^ ^10^, ^11^ ^]^ Much of the research in this field has focused on designing reversible transformations between 2D and 3D configurations using LCEs, due to mature 2D technology that easily arranges the liquid crystal (LC) orientation of different positions in the plane.^[^ ^24^, ^25^ ^]^ In contrast, 3D‐to‐3D deformation structures based on LCEs are capable of providing greater flexibility in shape transformation, making them more suitable for soft robotic systems that require multimodal deformation.^[^ ^26^, ^27^ ^]^ However, these efforts have encountered significant challenges in achieving intricate and complex 3D‐to‐3D transformations, primarily due to the inherent limitations of existing techniques for curvature‐driven deformation between 3D surfaces.

3D‐to‐3D deformation structures based on LCEs typically have an initial 3D as‐fabricated configuration, which was created using methods such as 3D printing,^[^ ^28^, ^29^, ^30^ ^]^ residual stress‐induced assembly,^[^ ^31^, ^32^ ^]^ and mechanical deformation‐induced assembly.^[^ ^33^, ^34^, ^35^, ^36^, ^37^ ^]^ Direct ink writing 3D printing is capable of directionally aligning LC molecules and spatially stacking multilayered filaments, making it highly convenient for the fabrication of 3D LCE structures with 3D‐to‐3D shape morphing.^[^ ^28^, ^29^ ^]^ However, it's hard to directly produce hollow or curved surface structures without the use of sacrificial supports. In contrast, the residual stress‐induced assembly^[^ ^31^, ^32^ ^]^ and mechanical deformation‐induced assembly^[^ ^33^, ^34^, ^35^, ^36^, ^37^ ^]^ can effectively fabricate curved 3D surface LCE structures through the transformation of 2D LCE films. For instance, methods such as annealing^[^ ^31^ ^]^ or evaporating^[^ ^32^ ^]^ residual stress, compression buckling,^[^ ^33^ ^]^ or template molding^[^ ^34^, ^35^, ^36^, ^37^ ^]^ can induce strip‐shaped LCE films to assemble into curved bands, including arc, wave, and spiral forms. By aligning the LC molecules before or during the assembly deformation, these structures can undergo reversible 3D shape transformations in response to external stimuli, such as arc‐to‐arc^[^ ^31^, ^32^, ^33^, ^34^, ^36^, ^37^ ^]^ or spiral‐to‐spiral.^[^ ^35^ ^]^ Despite significant efforts in this area, challenges remain in creating 3D LCE structures with nonzero‐Gaussian curvature^[^ ^38^ ^]^ and complex 3D‐to‐3D shape direct morphing. Current methods struggle to provide precise control over local curvature and directional deformation in the plane, making it difficult to customize and optimize the transformation of sheet‐shaped LCE films for specific applications. Overcoming these limitations is crucial for advancing the customization and programmability of LCE‐based 3D structures.

**Figure 1:**
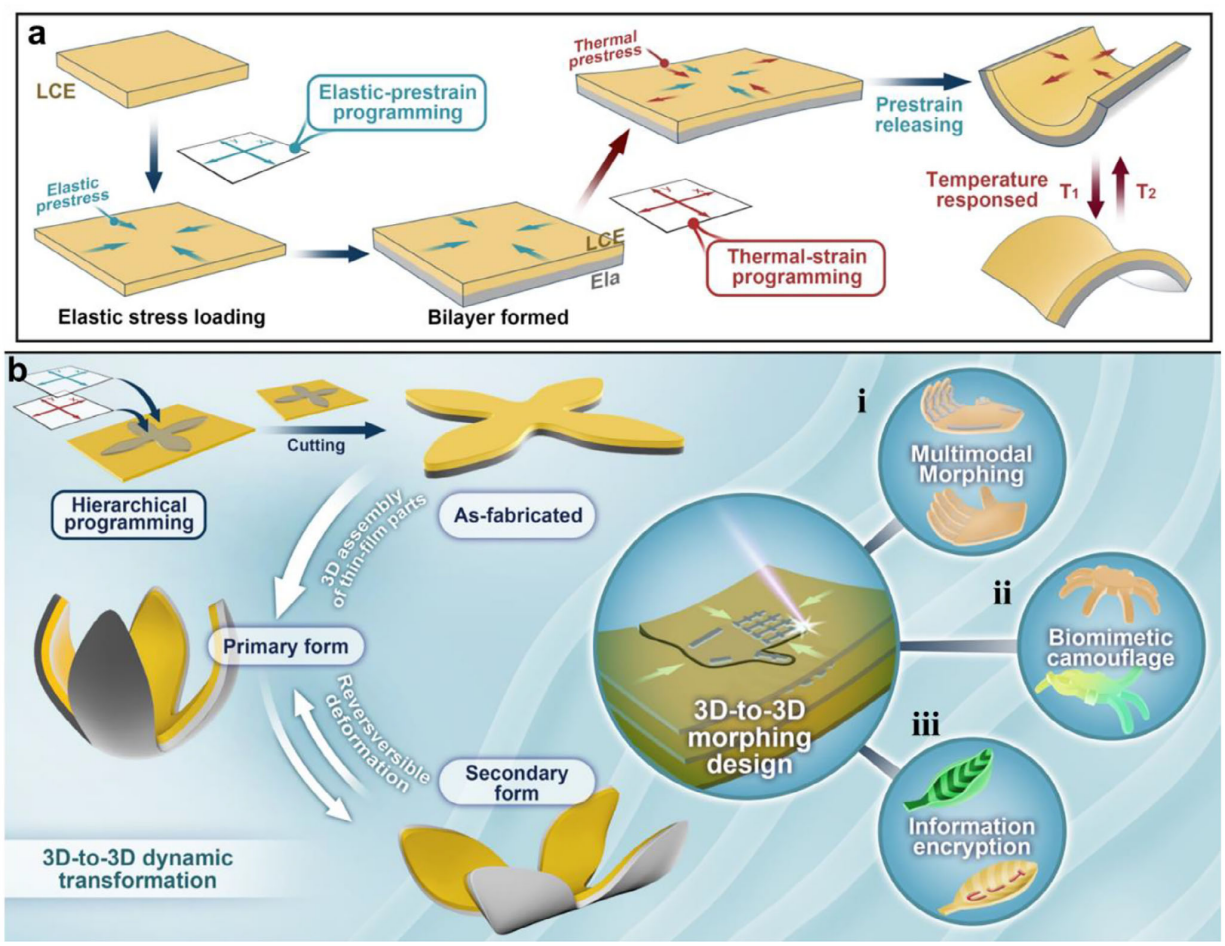
Schematic illustration of the mechanism and programming of hierarchical multimodal morphing. a) The two hierarchical transformations are achieved by pre‐stretched strain from the LCE‐Ela bilayer and thermal strain in LCE, respectively. The pre‐stress within the LCE layer is released, causing the bilayer film to deform into 3D structures, and then, through thermal actuation, this 3D structure undergoes further transformation into other 3D configurations driven by the anisotropic thermal deformation of the LCE material. b) The patterned mismatch strain and the application of pre‐strain and thermal strain in specific orientations are carried out to achieve the programmable desired 3D LCE‐Ela structures and multimodal 3D shape morphing i). Furthermore, this strategy easily creates vivid 3D LCE‐Ela structures with 3D shape transformations and color changes through coating thermochromic ink, used for biomimetic camouflage ii) and information encryption iii).

In this work, we present a simple strategy for creating 3D LCE‐Ela bilayer structures capable of achieving customized complex curvatures through a hierarchical shape transformation process (**Figure** **1**). The first step enables the transformation from a single 2D initial configuration into a 3D structure with complex curvature. The second step allows for the thermally actuated transformation of this 3D complex curvature into another distinct 3D configuration, enabling multimodal shape morphing. The two hierarchical transformations are achieved by pre‐stretched strain from the LCE‐Ela bilayer and thermal strain in LCE (Figure 1a), respectively. Upon removing constraints, the pre‐stress within the LCE layer is released, causing the bilayer film to deform into different 3D complex structures depending on the direction of pre‐stress and the printing pattern; through thermal actuation, each deformed structure undergoes further multimodal transformation into various 3D configurations, driven by the anisotropic thermal deformation of the LCE material. To achieve the programmable desired 3D LCE‐Ela structures (Figure 1b) and multimodal 3D shape morphing (Figure 1b,i), we introduced a patterned mismatch strain by leveraging DLP to print preset patterns onto LCE films, followed by the application of pre‐strain and thermal strain in specific orientations. The reversible thermal deformation of LCEs under heating and cooling induces mismatch thermal strain, enabling the programmed 3D LCE‐Ela structures to undergo controllable 3D‐to‐3D shape morphing. Comprehensive experimental and computational studies of the reversible 3D‐to‐3D shape‐morphing behaviors in over 30 3D LCE‐Ela structures demonstrate the reliability of the proposed strategy. Furthermore, we create biomimetic 3D LCE‐Ela structures with vivid 3D shape transformations and color changes (Figure 1b,ii) through coating thermochromic ink, enabling them to exquisitely mimic adaptive behaviors in response to environmental changes. Inspired by biology, we also propose a leaf‐shaped 3D LCE‐Ela structure, which can respond to thermal stimuli, quickly deform, and discolor to reveal itself and convey information (Figure 1b,iii).

## 2 Results and Discussion

### 2.1 Hierarchical 3D Morphing from 2D LCE‐Ela Bilayer Procedures

**Figure 2:**
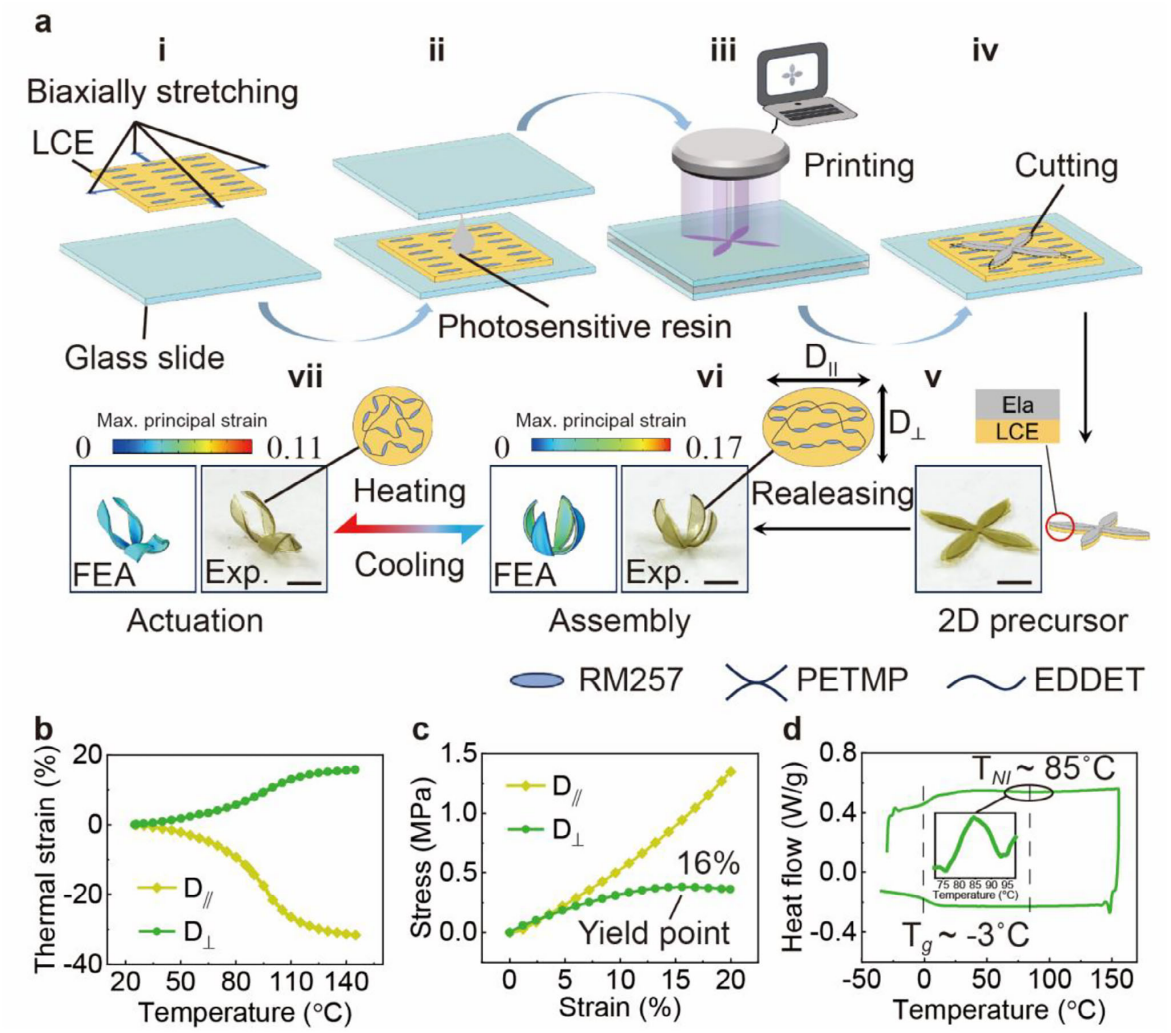
3D LCE‐Ela structures with 3D‐to‐3D hierarchical shape morphing strategy via mismatch strain induced by stress relaxation, and the mechanical and thermodynamic properties of the LCE films. a) Schematic illustration of the fabrication procedure of a representative 2D LCE‐Ela precursor and the reversible 3D‐3D shape‐morphing behavior of the 3D LCE‐Ela structures assembled from it: i) The pre‐stretched LCE films were fixed onto a glass slide. ii) Photosensitive resin was injected onto the films, and another glass slide was placed on top to form a reaction cell. iii) A pattern was projected onto the resin using DLP printing, curing a layer of Ela onto the LCE films. iv) The printed sample was cut around the cured pattern to obtain bilayer structures of LCE and Ela. v) a 2D precursor stuck on the glass substrate. Hierarchical shape morphing includes the assembly 3D morphing vi) induced by pre‐strain in LCE‐Ela bilayer by releasing the 2D precursor from the glass substrate, and actuation 3D morphing vii) induced by thermal strain in LCE‐Ela bilayer. b) The thermal strain‐temperature curves of the LCE films along directions parallel (*D*_∥_) and perpendicular (*D*_⊥_) to the alignment. c) The stress–strain curves of the LCE films along *D*_∥_ and *D*_⊥_. d) Differential scanning calorimetry measurement of the LCE films. All scale bar: 3 mm.

The hierarchical 3D Morphing procedure of a representative 2D LCE‐Ela bilayer structure is schematically illustrated in **Figure** **2**. The 2D‐to‐3D assembly is achieved by the mismatch strain induced between the active LCE substrate and the passive Ela layer, which is a result of pre‐stretching the active LCE substrate. And the subsequent 3D‐to‐3D reversible transformation was driven by the thermal anisotropic strain of the LCE material. The elastic and thermal behavior of the LCE layer was achieved by uniaxially aligned molecules, and was prepared following the two‐stage thiol‐acrylate Michael addition reaction methodology (detailed fabrication process in the Experimental Section). The Ela layer was formed by an elastic photosensitive resin, and was used as the passive components, ensuring that the 3D LCE‐Ela structures do not undergo irreversible plastic deformation during actuation. The fabrication process of 2D LCE‐Ela precursors is mainly divided into four steps and is detailly shown in Figure 2a. First, the pre‐stretched LCE films was first fixed onto the glass slide (Figure 2a,i). A drop of photosensitive resin then was injected onto the pre‐stretched LCE film, and a second glass slide was placed on top to ensure uniform coverage of the resin across the LCE surface. This setup formed a reaction cell for the subsequent photosensitive process (Figure 2a,ii). Furthermore, a designed pattern was projected onto the photosensitive resin in the reaction cell through DLP printing, which cured as a layer of Ela onto the LCE films after a given irradiation time (Figure 2a,iii). Finally, the printed sample was cut around the cured pattern to obtain the bilayer structure (2D precursor), consisting of an LCE and an Ela layer, with the thicknesses of 50 and 150 µm, respectively (Figure 2a,iv).

The hierarchical morphing procedures consist of two sequential stages: a 2D‐to‐3D transformation and a subsequent 3D‐to‐3D reconfiguration. After removal of 2D precursors from the glass slide, the unsymmetric pre‐strain of the bilayer along thickness results in a bending moment, and the 2D LCE‐Ela precursor (Figure 2a,v) spontaneously transforms into a stable 3D LCE‐Ela structure (Figure 2a,vi; Movie S1, Supporting Information). Then, the 3D LCE‐Ela structure was heated, which disrupts the LC molecular order and generates anisotropic thermal strain in the LCE, driving the 3D LCE‐Ela structure to transform further into another 3D configuration (Figure 2a,vii). The nematic‐to‐isotropic transition of the LCE substrates can be reversibly achieved by heating and cooling across the transition temperature, enabling macroscopic reversible 3D‐to‐3D deformation of the 3D LCE‐Ela structures (Figure 2a; Movie S2, Supporting Information). As verification, Finite Element Analysis (FEA) was also carried out, the shapes predicted by which agree very well with the experimental (Exp.) observations (Figure 2a,vi,vii).

The LCE is mechanically and thermally orthotropic, which will have a great influence on the final configuration of the bilayer. To analyze the anisotropic behavior, we characterized the mechanical and thermal properties of the LCE films along directions parallel (*D*_∥_) and perpendicular (*D*_⊥_) (Figure 2a) to the molecular orientation using a dynamic mechanical analyzer. Figure 2b presents the thermal strain‐temperature curves of the LCE films along *D*_∥_ and *D*_⊥_ directions. It is observed that molecular disorder induced by heating the uniaxially aligned LCE film leads to significant thermal contraction (≈34%) and expansion (≈17%) along *D*_∥_ and *D*_⊥_ directions, respectively. This opposite deformation behavior is a direct consequence of the thermal‐induced changes in the alignment of the orthotropic LCE molecules. Moreover, uniaxial tension tests measured at room temperature also show mechanical orthotropy of the LCE films, as shown in Figure 2c. The LCE films exhibit elastic behavior along *D*_∥_ direction, while it demonstrates elastic‐plastic characteristics along *D*_⊥_ direction, with a yielding strain of 16%. When the strain is small (less than 7%), the stress–strain curves in both directions nearly coincide. In this study, for simplicity, we disregard the mechanical anisotropy and only consider the anisotropic thermal strain of the LCE for the numerical simulation. The phase change behavior of LCEs was characterized by differential scanning calorimetry (DSC), as shown in Figure 2d. The glass transition temperature (Tg) of LCE is −4 °C, confirming that the material remains in an elastomeric state at room temperature and above, along with a pronounced phase transition from the nematic to isotropic phase at ≈85 °C (T_NI_).

### 2.2 Mechanism of Programmable 3D‐to‐3D Morphing Customization

**Figure 3:**
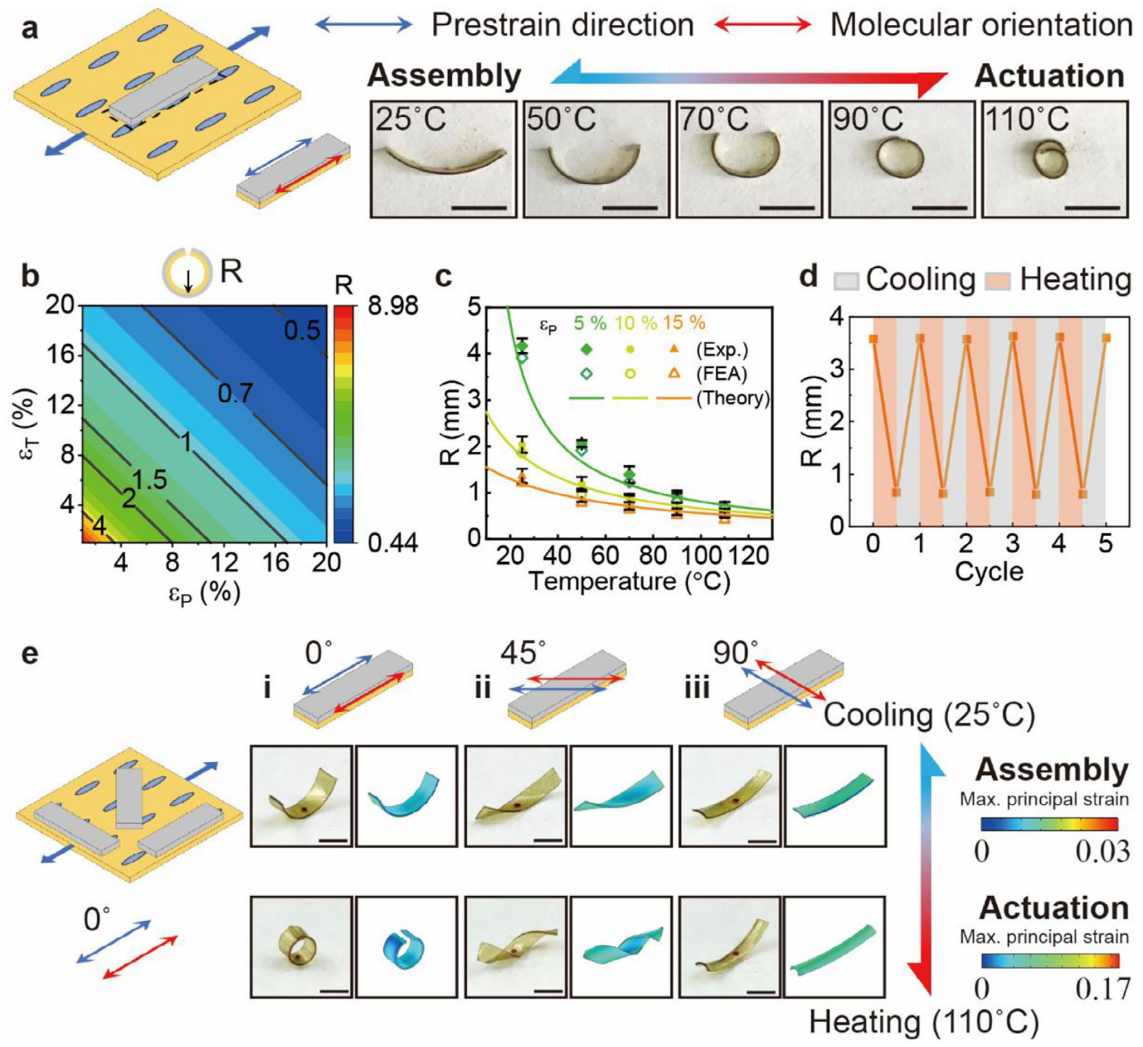
The effects of pre‐strain and thermal strain magnitude and distribution on the assembly and actuation shapes of the rectangular 2D precursor. a) The fabrication of a rectangular 2D precursor with the pre‐strain direction (blue arrow) and molecular orientation (red arrow) parallel to its longitudinal direction, and its shape at different temperatures. b) Contour plot of the curvature radius (R) in terms of the pre‐strain (ε_P_) and thermal strain (ε_T_). c) Exp., FEA, and theoretical results of the curvature radius for the arc‐shaped LCE‐Ela structure assembled with different pre‐strains (ε_P_ =  5%,  10% and 15%) at different temperatures. d) The variation in the curvature radius of the arc‐shaped LCE‐Ela structure under repeated heating (110 °C) and cooling (25 °C). e) The fabrication of three rectangular 2D precursors by printing Ela at different positions of pre‐stretching the LCE substrate along the direction parallel to the alignment (ε_P_ =  10%), and the FEA and Exp. results of their assembly and actuation shapes. All scale bar: 3 mm.

We explored the programmable and customizable transformation mechanisms of 3D LCE‐Ela structures using a rectangular strip precursor (10 mm × 2 mm), as shown in **Figure** **3**. The pre‐strain (blue arrow) is parallel to the molecular orientation (red arrow), and the longitudinal direction of the rectangular varies to achieve different configurations. Figure 3a shows the deformation of the structure when the longitudinal direction coincides with the pre‐strain and molecular orientation. The applied pre‐stretch is ε_P_ =  5% and arc‐shaped structure forms as assembled, whose curvature increases with increasing temperature. The unsymmetric pre‐stretch of the bilayer along thickness induced the bending moments parallel to the length direction, resulting in the rectangular 2D LCE‐Ela precursor bends to assemble into the arc‐shaped LCE‐Ela structure. Due to the alignment between the pre‐strain direction and molecular orientation (thus thermal stain direction), the thermal shrinkage of the LCE substrate upon heating generates a bending moment along the same direction as before, driving the arc‐shaped LCE‐Ela structure with the curvature radius 1.36 mm (25 °C) to bend further and eventually transform into a ring shape with the curvature radius 0.56 mm (110 °C).

To understand the thermal‐mechanical behavior of the arc‐shaped LCE‐Ela structure, we developed a theoretical model (Theoretical Model of Thermal‐Mechanical Deformations in Supporting Information). For a bilayer structure with given geometrical and mechanical properties, the curvature radius, $R=\frac{t_{2}}{6(\varepsilon_{P}+\varepsilon_{T})}\;\left(\frac{1+\frac{E_{1}}{E_{2}}\frac{t_{1}}{t_{2}}\left(4+\frac{t_{1}}{t_{2}}\left(6+\frac{t_{1}}{t_{2}}\left(4+\frac{E_{1}}{E_{2}}\frac{t_{1}}{t_{2}}\right)\right)\right)}{\frac{E_{1}}{E_{2}}\frac{t_{1}}{t_{2}}\left(1+\frac{t_{1}}{t_{2}}\right)}\right)$, depends only on the pre‐strain (ε_P_) and thermal strain (ε_*T*_) of the LCE substrate, where *E*_1_ and *t*_1_ of LCE, *E*_2_ and *t*_2_ of Ela are the Young's moduli and thicknesses, respectively. As shown Figure 3b, it is evident that the curvature radius decreases linearly with both increasing pre‐strain and thermal strain. As validation, we compare the curvature radius (R) of the arc‐shaped LCE‐Ela structure with different pre‐strains (ε_P_ =  5%,  10% and 15%) at different temperatures obtained by the theoretical model, FEA, and Exp. in Figure 3c. Consistent with previous analysis, the curvature of the arc‐shaped LCE‐Ela structure assembled from the 2D precursors with higher pre‐stretch is greater, and the thermal shrinkage of the LCE substrate induced by heating will further increase their curvature. The FEA results align well with the Exp. and theoretical model data, which ensures the validity of using FEA to predict the assembly or actuation deformation of other shape 2D precursors. Furthermore, we evaluated the reversibility and stability of the 3D LCE‐Ela structure change by measuring the curvature radius through a cycling test (Figure 3d). After 5 repeated heating via a heat gun and cooling via natural convection, the curvature radius of the arc‐shaped LCE‐Ela structure stably fluctuates between 3.7 mm (110 °C) and 0.6 mm (25 °C), without degradation of its shape occurring.

To achieve more complex 3D morphing, the angle between the rectangular direction, pre‐strain direction, and molecular orientation varies. In Figure 3e, the angle between the strip longitudinal and the molecular orientation varies from 0° to 90°. The LCE substrate was pre‐stretched to ε_*P*_ =  10% and three rectangular strips precursors were printed and cut with different longitudinal orientations. The pre‐strain direction (blue arrow) and molecular orientation (red arrow) of the LCE substrate remain parallel as in the previous case, and the angle between the strip longitudinal direction and the molecular orientation varies (0°, 45°, 90°). Accordingly, their bending moment direction for assembling and actuating will also change, generating three distinct 3D LCE‐Ela structures such as arc (Figure 3e, i, iii) and spiral (Figure 3e, ii), along with corresponding 3D‐to‐3D shape morphing (Movie S3, Supporting Information) such as arc‐to‐circle (Figure 3e, i), spiral‐to‐spiral (Figure 3e, ii), and arc‐to‐saddle (Figure 3e, iii). Furthermore, the pre‐strain directions (blue arrow) and molecular orientation (red arrow) can also be independently adjusted, adding one more freedom to morphing the structure. Modulating rectangular direction with various combinations of pre‐strain directions and molecular orientations, multiple complicated 3D‐to‐3D shape transformations can be achieved, as arc‐to‐spiral (i, iii, vi, ix), spiral‐to‐circle (ii, x), arc‐to‐circle (iv), spiral‐to‐spiral (v), arc‐to‐saddle (vi), in Figure S4 and Movie S3 (Supporting Information). Specifically, in the 3D deformation of rectangular‐strip precursors, all shapes except for the arc and circle exhibited nonzero Gaussian curvature. It is worth noting that the relative relationship between the pre‐strain orientation and molecular orientation of 2D precursors prepared from the same stretched LCE substrate is invariant regardless of the print position. As in the previous conclusion (Figure 2b, c), the bending degree in the assembly shapes of these precursors with varying pre‐strain directions and molecular orientations can also be further adjusted by the pre‐strain (Figure S5, Supporting Information). The assembly and actuation configurations predicted by FEA show great agreement with the Exp. results. These results suggest that by adjusting the printing position of the Ela, strategic distributions of pre‐strain and thermal strain can be introduced into the LCE substrate of the 2D LCE‐Ela precursor, enabling the assembly and actuation shapes to be programmed in a deterministic manner. More interestingly, the bending moment can be deflected by printing parallel‐strip Ela on LCE films, which also affects the assembly and actuation shape of the rectangular 2D precursors (Figure S6, Supporting Information). This observation leads us to investigate the potential for further diversifying and enabling multimodal 3D deformations through strategic design of the precursor shapes.

### 2.3 Multimodal 3D Transformations with Non‐Zero Gaussian Curvature

**Figure 4:**
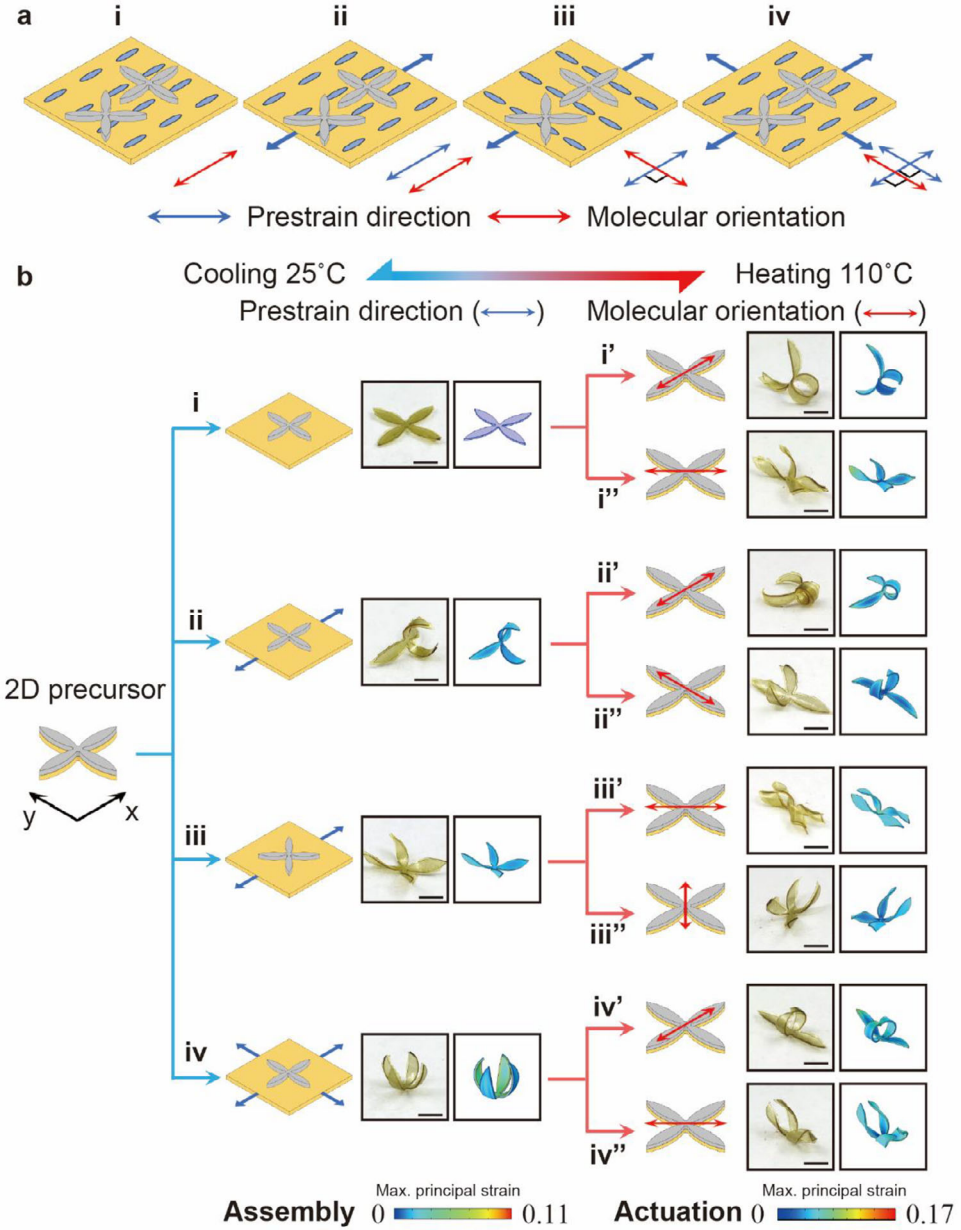
A design strategy to achieve customizable 3D shape transitions in different directions for the flower‐shaped 3D LCE‐Ela structures. a) The fabrication of a flower‐shaped 2D precursor with eight different pre‐strain directions (blue arrow) and molecular orientations (red arrow), achieved by combining four stretching methods with two printing configurations. b) The design routine, the FEA, and Exp. results of the assembly and actuation shapes of the eight flower‐shaped 2D precursors. All scale bar: 3 mm.

To further explore the multimodal deformations, we extend 2D precursors fabrication technique from strips to patterned sheets, exploring its ability to morph surfaces with non‐zero Gaussian curvature. Using the flower‐shaped 2D precursors as an example, we presented a design strategy to achieve customizable non‐zero Gaussian curvature 3D shape transitions in different directions for the 3D LCE‐Ela structures (**Figure** **4**; Movie S4, Supporting Information), through adjusting the direction of the pre‐strain relative to the thermal strain in the LCE substrate. Figure 4a illustrates the fabrication of one flower‐shaped 2D precursors with different pre‐strain directions (blue arrow) and molecular orientations (red arrow), achieved multimodal 3D‐to‐3D transformations by combining four stretching methods (unstretched (Figure 4a,i), stretched along parallel (Figure 4a,ii) and perpendicular (Figure 4a,iii) directions to the molecular orientation, and biaxial stretched (Figure 4a,iv)) with two printing configurations. Figure 4b shows the hierarchical design strategy for customizable shape 3D‐to‐3D transitions in different directions, along with the FEA and Exp. results of the assembly and actuation of multimodal shapes from the flower‐shaped 2D precursors. Here, we establish a planar local Cartesian coordinate system (x‐y) to facilitate the description of the two directions of the petals as shown in Figure 4b. First, the flower‐shaped 2D precursors prepared from the unstretched LCE substrate without pre‐strain (Figure 4a,i) remain flat at room temperature (Figure 4b,i). Upon heating, the anisotropic thermal deformation of the LCE substrate causes the petals of the x and y directions to bend (Figure 4b,i) or twist (Figure 4b,i) in opposite directions. By combining stretching in different directions with printing Ela at various positions, the petals along the x‐ and y‐direction can be independently guided to the different assembly shapes. For example, the flower‐shaped 2D precursors prepared with different orientations (Figure 4a,ii,iii) can form the assembly shapes with the petals that exhibit opposite bending (Figure 4b,ii) or twisting (Figure 4b,iii) along the x‐ and y‐direction. In comparison, the biaxially stretching LCE substrate undergoes isotropic elastic deformation, causing the flower‐shaped 2D precursors prepared from it (Figure 4a,iv) to form the assembly shapes with petals that bend in the same direction (Figure 4b,iv). Furthermore, due to the anisotropic thermal deformation of the LCE substrate, the bending or twisting petals of these 3D LCE‐Ela structures can morph into the 3D configurations of the oppositely bending (Figure 4b,ii,iv) or twisting (Figure 4b,iii,iii,iv) along the x‐ and y‐direction upon heating. By combining the above hierarchical morphing strategies, it becomes possible to design multimodal 3D‐to‐3D shape transitions with non‐zero Gaussian curvature in different directions for arbitrary LCE‐Ela structures. The resulting Exp. 3D shapes closely agree with the FEA simulation, further validating the versatility of the proposed strategy.

**Figure 5:**
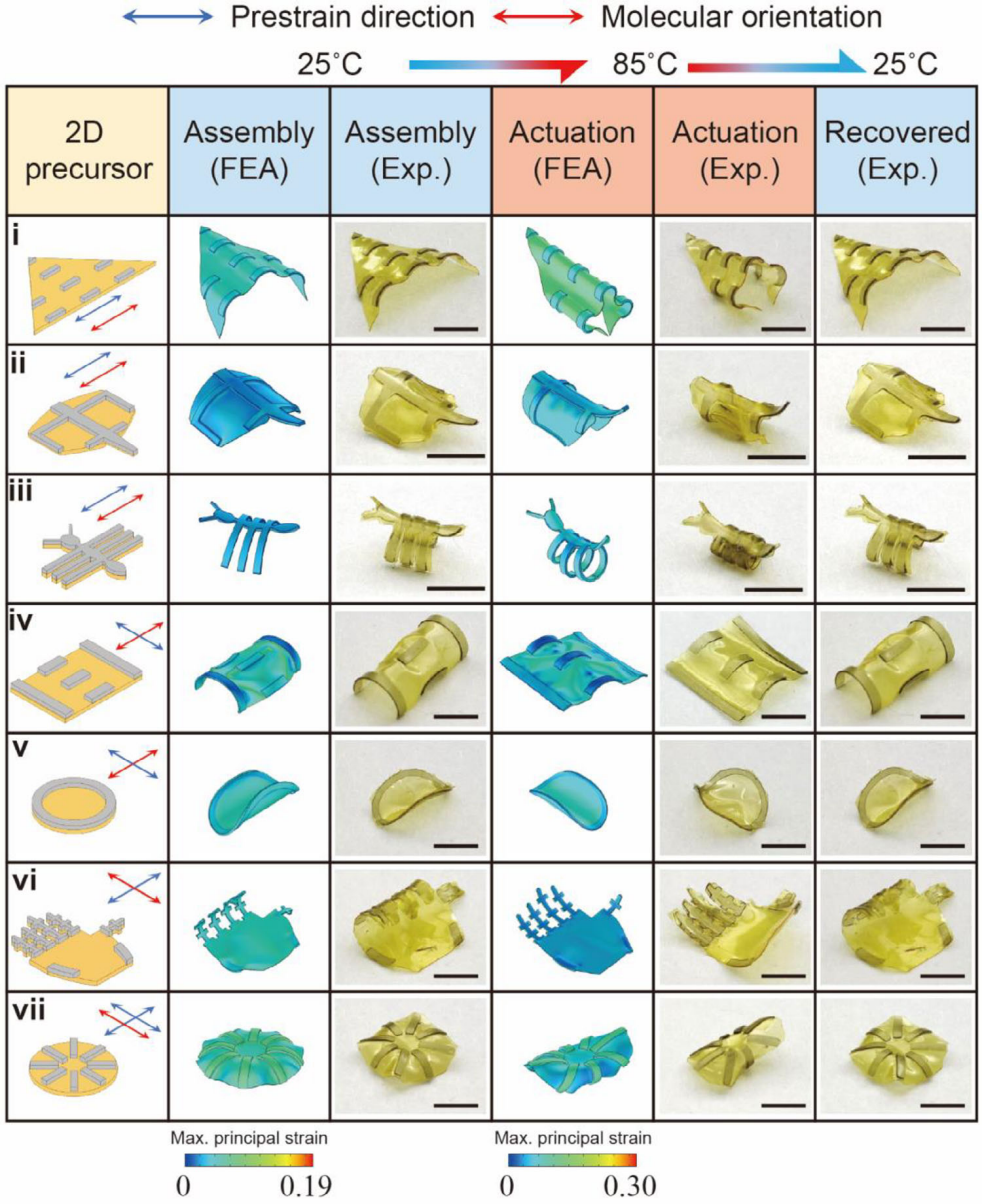
The Exp. results and FEA predictions for the multiple‐curved 3D LCE‐Ela structures formed from the pattern‐designed 2D precursors with varying pre‐strain directions (blue arrow) and molecular orientations (red arrow), as well as the reversible 3D‐to‐3D shape transformations: i) a wing, ii) a manta iii) an ant, iv) a massif, v) a burrito, vi) a palm, vii) a parachute. All scale bar: 5 mm.

Owing to the ease of access and customizability, by combining programmed pre‐strain and thermal strain distribution with the pattern‐designed in plane, we further presented the multiple‐curved 3D LCE‐Ela structures (varied Gaussian curvature) with complex 3D‐to‐3D shape morphing (Movie S5, Supporting Information). **Figures** **5** and S7 (Supporting Information) illustrate Exp. results and FEA predictions for a collection of 3D LCE‐Ela structures form reversible 3D‐to‐3D shape transformations from the pattern‐designed 2D precursors with varied pre‐strain directions (blue arrow) and molecular orientations (red arrow). The pattern‐designed 2D precursor and the assigned pre‐strain direction enable the local curvature of assembly morphing in a controlled manner, thereby creating a series of multiple‐curved 3D structures, including shapes as a wing (Figure 5,i), a manta (Figure 5,ii), an ant (Figure 5,iii), a massif (Figure 5,iv), a burrito (Figure 5,v), a palm (Figure 5,vi), a parachute (Figure 5,vii) and a mask (Figure S7, Supporting Information). Though combining the careful design of the molecular orientation of the LCE substrate, this series of multiple‐curved 3D structures can demonstrate vivid 3D‐to‐3D shape deformations, such as wing folding (Figure 5,i), pectoral fin flapping (Figure 5,ii), body curling (Figure 5,iii), tectonic movement (Figure 5,iv), opening and closing (Figure 5,v), finger spreading (Figure 5,vi), curling (Figure 5,vii), and mask changing (Figure S7, Supporting Information). The demonstrated structures with nonzero‐Gaussian curvature and complex 3D‐to‐3D shape morphing are very challenging to achieve with existing techniques such as 3D printing, compression buckling, or template molding. As a result, such a strategy greatly expands the available classes of 3D‐to‐3D deformation structures based on LCEs. Furthermore, this approach is expected to facilitate the creation of more complex 3D LCE structures with 3D‐to‐3D shape transformations, due to the adjustability of the pre‐strain and thermal strain distributions in the LCE substrate, combined with the geometry‐design flexibility of the passive Ela.

### 2.4 3D‐to‐3D Morphing Strategy for Biomimetic Applications

**Figure 6:**
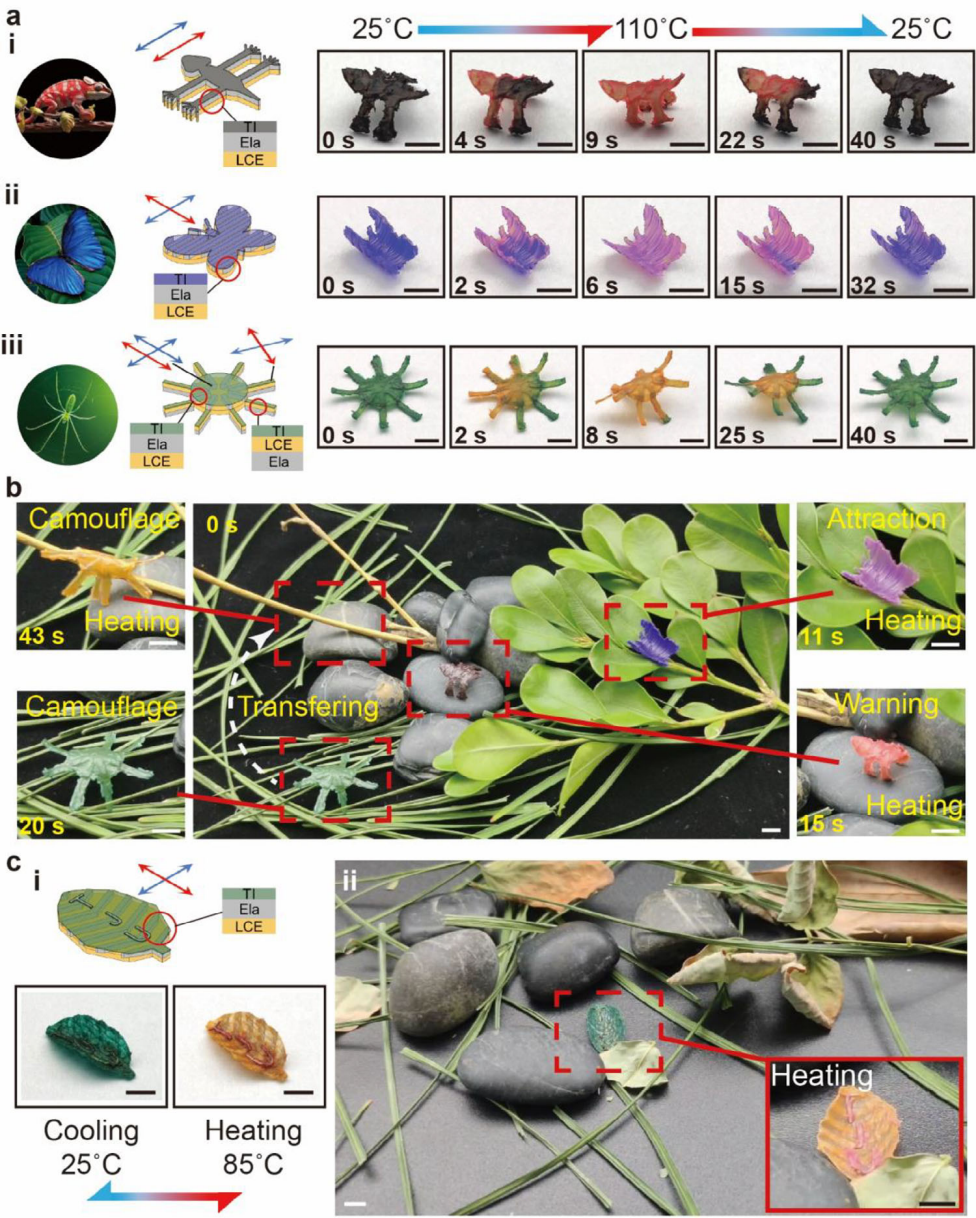
Biologically inspired to a proof‐of‐concept biomimetic application of 3D LCE‐Ela structures with dynamically simultaneous deformation and discoloration. a) The 2D precursors of the three biomimetic 3D LCE‐Ela structures, including the chameleon i), butterfly ii), and spider iii), along with their reversible 3D‐to‐3D shape transformations and color changes at different times during heating and cooling. b) Placed these 3D biomimetic structures in a real natural environment to display their adaptive deformation and discoloration, driven by a heating stimulus. c) The 2D precursors i), and shape and color at different temperature of the leaf‐shaped 3D LCE‐Ela structure capable of information encryption and camouflage; ii) The leaf‐shaped 3D LCE‐Ela structure blends with its environment, camouflaging itself, but deforms and discolors when heated to reveal hidden information, returning to its original form and color once the heat was removed. All scale bar: 5 mm.

Biological systems can dynamically control their color and shape, enabling them to perform functions such as warning, courtship, and camouflage, to survive in harsh and diverse environments. Inspired by these biological behaviors, we here demonstrate a proof‐of‐concept biomimetic application of 3D LCE‐Ela structures with dynamically simultaneous deformation and discoloration based on the hierarchical 3D‐to‐3D morphing strategy (**Figure** **6**). To incorporate the thermochromic discoloration capability into the 3D LCE‐Ela structures, we applied a thin layer of thermochromic ink (TI, thickness less than 10 µm), which undergoes reversible color changes in response to temperature (≈45 °C), onto the surface of the 2D LCE‐Ela precursors (detailed fabrication process in the Experimental Section). Figure 6a shows the 2D precursors of the three biomimetic 3D LCE‐Ela structures, including the chameleon (Figure 6a,i), butterfly (Figure 6a,ii), and spider (Figure 6a,iii), along with their reversible 3D‐to‐3D shape transformations and color changes at different times during heating and cooling. To achieve life‐like biomimetic 3D deformation and discoloration, the design of the printing patterns, pre‐strain direction, molecular orientation, and thermochromic inks for each 2D precursor is tailored according to the behavioral characteristics of these organisms, as shown in Figure 6a. Driven by a heat gun (110 °C), these biomimetic 3D LCE‐Ela structures can rapidly change their shape and color in 6–8s. The chameleon‐shaped LCE‐Ela 3D structure will close its limbs while changing from brown to red (Figure 6a,i; Movie S6, Supporting Information); the butterfly‐shaped LCE‐Ela 3D structure can spread wings while changing from blue to purple (Figure 6a,ii; Movie S6, Supporting Information); the spider‐shaped LCE‐Ela 3D structure will raise its body and change from green to orange (Figure 6a,iii; Movie S6, Supporting Information). However, it takes longer (≈32–40s) for these structures to recover its initial 3D configurations due to the relatively slow natural convection. The exceptional ability of vivid 3D‐to‐3D shape transformations and color changes enables these 3D biomimetic structures to exquisitely mimic the adaptive behaviors of natural organisms in response to environmental changes. As shown in Figure 6b, we placed these 3D biomimetic structures in a real natural environment to display their adaptive deformation and discoloration (Movie S6, Supporting Information), driven by a heat gun. The butterfly resting on the tree flutters flapped its wings, and changed color within 11 s to attract potential mates (Figure 6b, top right). When encountering an enemy, the chameleon standing on the rock curls its body into an aggressive posture while transforming its body into a striking red color within 15 s to signal a warning (Figure 6b, bottom right). The spider showcases multiple adaptive camouflage abilities. Upon landing on the grass, it extends its green legs outward to seamlessly blend with the surrounding weeds within 20 s (Figure 6b, bottom left). Once transferred to a tree trunk, the spider swiftly adjusts its posture and color, effectively camouflaging itself as a yellow branch within 43 s (Figure 6b, top left). Compared to previously reported synergistic color and shape changes in flat structures,^[^ ^39^, ^40^, ^41^ ^]^ the 3D LCE‐Ela structures presented here exhibit reversible life‐like 3D‐to‐3D shape transformations and corresponding color changes that more closely align with real biological behaviors. Our strategy holds great potential for developing artificial biomimetic devices and soft actuators with multifunctional capabilities, integrating synergistic discoloration and 3D deformation.

Another purpose of the color and shape changes in the nature organisms is to convey information to the outside world. Inspired by this, we have developed a leaf‐shaped 3D LCE‐Ela structure that has the ability to information encryption and camouflage, as shown in Figure 6c (Movie S7, Supporting Information). The leaf‐shaped 2D precursors consist of an LCE substrate (Figure 6c,i) with pre‐strain directions perpendicular to the molecular orientations, a printing pattern in the shape of leaf veins, a thin layer of thermochromic ink (green‐to‐orange), and three letter‐shaped papers coated by thermochromic ink (green‐to‐pink). Under heating, the green curled leaf‐shaped 3D LCE‐Ela structure transforms into orange and unfolds the leaves to expose the three pink letter‐shaped papers in its surface (Figure 6c,i). This leaf‐shaped 3D LCE‐Ela structure can be scattered randomly in the woods (Figure 6c,ii). Its initial 3D leaf‐like shape with a green appearance allows it to blend seamlessly into the surrounding environment, providing effective camouflage to avoid detection. Furthermore, the color of the letters closely matches that of the leaves, ensuring the surface information remains well concealed. When heated to 85 °C, the leaf‐shaped 3D LCE‐Ela structure undergoes rapid deformation and discoloration, making it strikingly visible and revealing hidden information. Once the heat stimulus is removed, it returns to its original shape and color, resuming its camouflage and concealing the information again. Compared to previous 2D‐shaped encryption systems,^[^ ^42^, ^43^ ^]^ such proposed biomimetic 3D LCE‐Ela structure integrating deformation and discoloration demonstrates superior concealment capabilities.

For biomimetic camouflage and information encryption, rapid response (< 10 s) and reversible cycling are critical. Our structures achieve deformation/discoloration in 6–8 s (Figure 6a), with > 5 reversible cycles (Figure 3d). Furthermore, the programming strategy enables application‐specific customization. For example, adjusting local pre‐sretrain orientations mimics biological movements (Figure 6a), while thermochromic patterning creates environment‐responsive information encryption (Figure 6c). While thermal actuation ensures stability across 25–110 °C (Figure 2d), humidity/light effects will be explored in future work.

## 3 Conclusion

In summary, we introduced a simple and efficient hierarchical approach to design 3D LCE‐Ela structures with customizable complex curvatures derived from 2D precursors, then enabling 3D‐to‐3D multimodal shape transformations with enhanced versatility. This strategy introduces interfacial mismatch strain between the LCE and Ela layers through the release of pre‐strain, thermal strain, and pre‐designed pattern in the bilayer. By using DLP to print preset patterns, combined with different pre‐strain and thermal strain orientations, we can precisely control the desired 3D LCE‐Ela structures and 3D‐to‐3D multimodal shape morphing. The Exp. and FEA results from this study showed the feasibility of over 30 different configurations of 3D LCE‐Ela structures, including arc, spiral, and biomimetic shapes, and demonstrated their repeatability in multiple deformations. Moreover, by coating with thermochromic ink, we successfully achieved biomimetic 3D LCE‐Ela structures with shape transformations and color changes, mimicking adaptive behaviors, such as courtship, aggression, and camouflage, in response to environmental changes. Inspired by biology, we also designed a leaf‐shaped 3D LCE‐Ela structure that quickly deforms and changes color in response to thermal stimuli, thereby conveying information. Our method presents a straightforward method to address the gap in 3D‐to‐3D complex shape morphing, offering a customizable approach for generating arbitrary 3D shapes with varied Gaussian curvatures. This work overcomes inherent limitations in existing techniques by achieving controllably customized local curvature and directional deformation. This advancement offers new insights into the development of multifunctional, intelligently responsive LCE‐based 3D structures, which broaden potential applications in areas such as soft robotics and information security, providing versatile and innovative design possibilities.

## 4 Experimental Section

### Materials

All chemicals were sourced from commercial suppliers and used without any prior treatment. The chemical reagents used to fabricate the synthesis of the LCE films include RM257 (monomer, > 97.0%, HDC), PETMP (crosslinker, > 95.0%, BJV), EDDET (flexible chain extender, > 95.0%, Adamas), DPA (thermal catalyst, > 99.0%, Aladdin), BHT (thermal inhibitor, > 99.0%, Adamas), P1173 (photocatalyst, > 98.0%, Aladdin) and toluene (solvent, > 99.0%, SCRC). The passive layer used for printing on the LCE films was a photosensitive resin (F80, Phrozen) that could be cured by UV light to a solid elastomer. For the thermochromic layer, a reversible thermochromic ink (produced by Hangzhou Zhicheng Technology) was employed, which would change color when heated above 45 °C (Figure S8, Supporting Information).

### Synthesis of the LCE Film

The fabrication process for the LCE films followed the two‐stage thiol‐acrylate Michael addition reaction methodology based on a previously reported recipe (Figure S9, Supporting Information).^[^ ^44^, ^45^ ^]^ The mixture of RM257 (676.9 mg), BHT (10 mg), and toluene (780.7 mL) was heated to 80 °C to ensure complete dissolution. After cooling to room temperature, PETMP (61.1 mg), EDDET (136.7 mg), and P1173 (16.75 mg) were added, and the solution was stirred for ≈10 min. This was then combined with a diluted DPA solution (3.35 mg, 1:50 w/w) and immediately injected into a reaction cell consisting of two quartz glass slides (50 mm × 50 mm × 1 mm) to undergo the first‐stage Michael addition reaction in the dark for ≈12 h, resulting in swollen polydomain LCE films. The films were subsequently placed in a vacuum chamber at 80 °C for 12 h to fully evaporate the toluene. The dried polydomain LCE films were stretched 50% of their original length, forming clear monodomain LCE films, which were then exposed to 365 nm UV light for the second‐crosslinking reaction to stabilize the alignment. The final monodomain LCE films (all thicknesses of 150 µm) were collected and used as the pre‐stretched substrate. The fabrication procedure and the final experiment sample of LCE films was schematically illustrated in Figure S10 (Supporting Information). Optical images of square LCE film were captured at different temperatures (Figure S11a, Supporting Information) and cross‐polarized optical images of square LCE film at different angle (Figure S10b, Supporting Information) to demonstrate its anisotropic properties. Furthermore, through thermal cycling tests, the stability of LCE's thermally actuated deformation was confirmed (Figure S11c, Supporting Information).

### Fabrication of LCE‐Ela 2D Precursors

The fabrication procedure of 2D precursors was schematically illustrated in Figure 1a. Using adhesive tape to attach the four edges of the LCE films for stretching, meanwhile the pre‐stretching LCE was fixed in a glass slide (Figure S12a, Supporting Information). A drop of photosensitive resin was injected onto the surface of the pre‐stretched LCE, and a glass slide was placed on top, forming a reaction cell (separated by spacers with a thickness of 200 µm). Then, the reaction cell was placed on a fixed platform positioned at the focal plane of a light projector (Photon mono 4K, Anycubic, wavelength 405 nm). A designed pattern was projected onto the photosensitive resin under a given irradiation (Figure S12b, Supporting Information). The continuous light triggered the photopolymerization process, resulting to print a layer of solid elastomer on the LCE films (Figure S12c, Supporting Information). Through process optimization, the optimal irradiation time was determined to be 7.5 s (Figure S13, Supporting Information). The entire process was conducted in a dark environment to avoid undesired polymerization before use. After printing, the top glass slide was removed, and the excess liquid resin was washed off with methanol. The clean sample was then dried in a vacuum oven at 25 °C for 15 min. Finally, the sample was cut around the curing pattern to obtain 2D precursors consisting of LCE (all thicknesses of 50 µm) and Ela (all thicknesses of 150 µm). In addition, by applying different irradiation times to the LCE‐Ela 3D structure (Figure S14, Supporting Information), its temperature and shape remained stable during the irradiation process, was confirmed. The interfacial microstructure after multiple thermal cycles (Figure S15a, Supporting Information) and peel testing (Figure S15b, Supporting Information) confirm the good interfacial bonding of the fabricated bilayer structure.

### Fabrication of LCE‐Ela 2D Precursors with Thermochromic Ink

The fabrication process of the 2 LCE‐Ela 2D precursors with thermochromic ink was the same as described above. Before the 2D precursors was released from the glass slide, the thermochromic ink was evenly applied to its surface using a brush, then left to dry and cure at room temperature to form the 2D precursors consisting of three layers of thermochromic ink, Ela, and LCE.

### Thermal Actuation of LCE‐Ela 3D Structures

The thermal actuation of the 3D structures was carried out by gradually heating them on a thermal platform. To ensure uniform heating, the 3D structures were covered with a transparent, closed cover positioned above them. Additionally, to facilitate rapid cooling after heating, a heat gun was used to induce actuation deformation in the thermochromic 3D structures. In addition to using a heat gun, it also successfully achieved the photothermal deformation of the LCE‐Ela 3D structure by coating a layer of black carbon powder on the surface and applying continuous infrared light irradiation (Figure S16, Supporting Information).

### Characterization

All optical images in the paper were captured using a digital camera (D90, Nikon). The phase transition temperatures of the LCE films were determined via differential scanning calorimetry (DSC, Q2000, TA Instruments). Using a dynamic mechanical analyzer (MCR102e, Anton Paar) to characterize the stress–strain relationship and thermal expansion properties of LCE films, and the stress–strain relationship and viscoelastic properties of Ela films. The curvature radius of the arc‐shaped LCE‐Ela structure was measured using ImageJ software.

### Finite Element Analysis

A quasi‐static analysis in FEA was conducted to simulate the morphing behavior of the assembly of 2D precursors and the actuation of 3D structures. Both the Ela and the LCE were modeled using eight‐node 3D solid elements. Refined meshes were employed to ensure computational accuracy. The elastic behaviors of the Ela and the LCE were captured using the Hookean model. By fitting the thermal strain‐temperature curve (Figure 2b) and stress–strain curve (Figure 2c), the material parameters of the LCE were determined, including the Young's modulus (isotropic, E_LCE_ = 4.48 mPa) and the coefficients of thermal expansion along the parallel alignment direction (α_∥_ = ‐0.0023 K^−1^) and the vertical alignment direction (α_⊥_ = 0.0011 K^−1^), respectively. The Ela was treated as an isotropic material (the testing of its mechanical and viscoelastic behavior is shown in Figure S17, Supporting Information), with its Young's modulus (E_Ela_ = 5 mPa) calibrated from Figure S17b (Supporting Information). Through dynamic mechanical analysis, it was confirmed that Ela remains elastic above room temperature (Figure S17c, Supporting Information). Initially, an applied strain was introduced to simulate the assembled deformation of the 2D precursor. Once the assembled shape stabilized, the actuated deformation of the 3D structures was simulated by gradually increasing the temperature.

## Conflict of Interest

The authors declare no conflict of interest.

## Author Contributions

J.T. performed the experiments, conceptualized the study, conducted formal analysis and methodology development, implemented the software, and wrote the original draft. C.S. contributed to funding acquisition and writing – review and editing. G.N. was involved in writing – review and editing. C.L. contributed to conceptualization, funding acquisition, provision of resources, and writing – review and editing. Y.Z. also contributed to conceptualization, funding acquisition, provision of resources, and writing – review and editing.

## Supporting information



Supporting Information

Supplemental Movie 1

Supplemental Movie 2

Supplemental Movie 3

Supplemental Movie 4

Supplemental Movie 5

Supplemental Movie 6

Supplemental Movie 7

## Data Availability

The data that support the findings of this study are available from the corresponding author upon reasonable request.;
