# Supplementary material for: Programmable and Reversible 3D‐to‐3D Shape Transformation: Hierarchical Multimodal Morphing Based on Liquid Crystal Elastomers
Source: Adv Sci (Weinh). 2025 Jul 28;12(40):e07922. doi: 10.1002/advs.202507922 (PMC12561347; doi:10.1002/advs.202507922)
Supplement: Supplementary file 1 — Supporting Information [file ADVS-12-e07922-s003.docx]

**Programmable and Reversible 3D-to-3D Shape Transformation: Hierarchical Multimodal Morphing Based on Liquid Crystal Elastomers**

Jiayu Tian^1^, Chuanqian Shi^2^, Guohua Nie^1^, Chenzhe Li^1^* and Ying Zhao^1^*

^1^School of Aerospace Engineering and Applied Mechanics, Tongji University, 100 Zhangwu Road, Shanghai 200092, China

^2^Center for Mechanics Plus under Extreme Environments, School of Mechanical Engineering & Mechanics, Ningbo University, Ningbo, China

**1.** **Theoretical Model of Thermal-Mechanical Deformations of 3D LCE-Ela Structure**

To simplify the model, the arc-shaped LCE-Ela structure is considered as a 2D bilayer beam structure (the thickness outside the surface is much smaller than the thickness and length within the surface), with the force diagram shown in Figure S1. The initial thickness of the LCE is $t_{1}$, and the initial thickness of the Ela is $t_{2}$. When stress relaxation and heating occur, the LCE is in compression, while the Ela is in tension, causing the bilayer to bend. Since no external force is applied to the beam, the forces and moments on the beam's cross-section must be in equilibrium, leading to the following conclusions:

$$\begin{aligned} F_{1}-F_{2}=0\#\left( S1 \right) \end{aligned}$$

$$\begin{aligned} M_{1}+M_{2}=F_{1}(\frac{t_{1}}{2}+\frac{t_{2}}{2})\#\left( S2 \right) \end{aligned}$$

Let the curvature radius of the neutral layer after the bilayer film bends be R. Considering that the change in the thicknesses of the bilayer films along the thickness direction is much smaller than the radius of curvature, it can be approximated that the curvature radius of both layers is the same. Therefore, the relationship between the bending moment of each layer and the radius of curvature R can be expressed as:

$$\begin{aligned} \left\{ \begin{matrix} M_{1}=\frac{E_{1}I_{1}}{R} \\ M_{2}=\frac{E_{2}I_{2}}{R} \end{matrix} \right.\#\left( S3 \right) \end{aligned}$$

Where, $E_{1}I_{1}$ and $E_{2}I_{2}$ represent the bending stiffnesses of the pre-stress layer and the cured layer, respectively, where $E_{1}$ and $E_{2}$ are the Young’s moduli of LCE and Ela. The thickness changes of the pre-stress and cured layers after deformation are relatively small and can be neglected. Therefore, it is assumed that their thicknesses remain unchanged before and after deformation, so their moments of inertia can be expressed as:

$$\begin{aligned} \left\{ \begin{matrix} I_{1}=\frac{t_{1}^{3}}{12} \\ I_{2}=\frac{t_{2}^{3}}{12} \end{matrix} \right.\#\left( S4 \right) \end{aligned}$$

Due to interface compatibility, the unit elongation at the contact surface of the two layers must be equal. Therefore:

$$\begin{aligned} \varepsilon_{P}+\varepsilon_{T}=\frac{F_{1}}{E_{1}t_{1}}+\frac{t_{1}}{2R}+\frac{F_{2}}{E_{2}t_{2}}+\frac{t_{2}}{2R}\#\left( S5 \right) \end{aligned}$$

Here, $\varepsilon_{P}$ represents the pre-strain, and $\varepsilon_{T}$ represents the thermal strain. By combining equations (S1), (S2), (S3), (S4), and (S5), an explicit analytical expression for the curvature radius can be obtained as:

$$\begin{aligned} R=\frac{t_{2}}{6(\varepsilon_{P}+\varepsilon_{T})}\left( \frac{1+\frac{E_{1}}{E_{2}}\frac{t_{1}}{t_{2}}\left( 4+\frac{t_{1}}{t_{2}}\left( 6+\frac{t_{1}}{t_{2}}\left( 4+\frac{E_{1}}{E_{2}}\frac{t_{1}}{t_{2}} \right) \right) \right)}{\frac{E_{1}}{E_{2}}\frac{t_{1}}{t_{2}}\left( 1+\frac{t_{1}}{t_{2}} \right)} \right)\#\left( S6 \right) \end{aligned}$$

Here, the thermal strain $\varepsilon_{T}$ can be expressed in terms of the relationship between the thermal expansion coefficient $\alpha$ and temperature:$\varepsilon_{T}=\alpha(T_{Heat}-T_{Room})$. Therefore, equation S6 can be rewritten as:

$$\begin{aligned} R=\frac{t_{2}}{6(\varepsilon_{P}+\alpha(T_{Heat}-T_{Room}))}\left( \frac{1+\frac{E_{1}}{E_{2}}\frac{t_{1}}{t_{2}}\left( 4+\frac{t_{1}}{t_{2}}\left( 6+\frac{t_{1}}{t_{2}}\left( 4+\frac{E_{1}}{E_{2}}\frac{t_{1}}{t_{2}} \right) \right) \right)}{\frac{E_{1}}{E_{2}}\frac{t_{1}}{t_{2}}\left( 1+\frac{t_{1}}{t_{2}} \right)} \right)\#\left( S7 \right) \end{aligned}$$

This model is applicable only to the analysis of bending deformation caused by thermo-mechanical coupling in the case of a bilayer thin film in two dimensions.

While the curvature radius R derived from beam theory is typically applied to simple planar models, it remains valid for determining principal curvatures in 3D bilayer deformations through directional strain decoupling. As shown in Figure S2, the two principal curvatures radius ($R_{1}$, $R_{2}$) are independently governed by strains along orthogonal principal directions ($d_{1}$, $d_{2}$):

1) Curvature radius ($R_{1}$) along pre-stretched direction ($d_{1}$) is expressed as:

$$\begin{aligned} R_{1}=\frac{t_{2}}{6(\varepsilon_{P}+\alpha(T_{Heat}-T_{Room}))}\left( \frac{1+\frac{E_{1}}{E_{2}}\frac{t_{1}}{t_{2}}\left( 4+\frac{t_{1}}{t_{2}}\left( 6+\frac{t_{1}}{t_{2}}\left( 4+\frac{E_{1}}{E_{2}}\frac{t_{1}}{t_{2}} \right) \right) \right)}{\frac{E_{1}}{E_{2}}\frac{t_{1}}{t_{2}}\left( 1+\frac{t_{1}}{t_{2}} \right)} \right)\#\left( S8 \right)\# \end{aligned}$$

2) By Poisson’s ratio $v$, Curvature radius ($R_{2}$) perpendicular to pre-stretched ($d_{2}$) is expressed as:

$$\begin{aligned} R_{2}=\frac{t_{2}}{6v(\varepsilon_{P}+\alpha(T_{Heat}-T_{Room}))}\left( \frac{1+\frac{E_{1}}{E_{2}}\frac{t_{1}}{t_{2}}\left( 4+\frac{t_{1}}{t_{2}}\left( 6+\frac{t_{1}}{t_{2}}\left( 4+\frac{E_{1}}{E_{2}}\frac{t_{1}}{t_{2}} \right) \right) \right)}{\frac{E_{1}}{E_{2}}\frac{t_{1}}{t_{2}}\left( 1+\frac{t_{1}}{t_{2}} \right)} \right)\#\left( S9 \right)\# \end{aligned}$$

As demonstrated in the comparison below than FEA (Figure S3), the two principal curvatures radius ($R_{1}$, $R_{2}$) derived from theoretical equations show close agreement with FEA simulations, confirming the validity of the calculation method of the two principal curvatures.


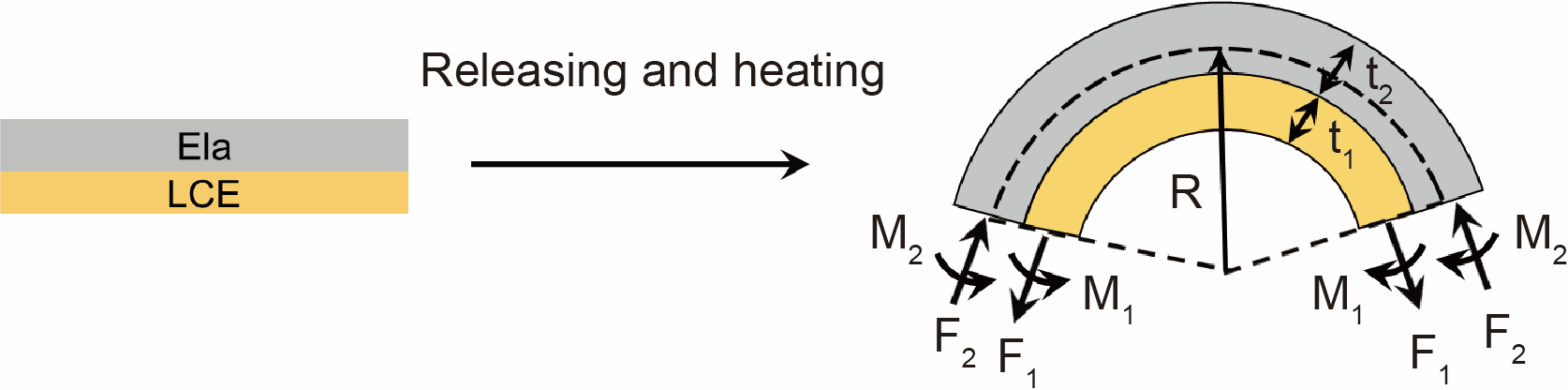


Figure S1. Theoretical model of the arc-shaped LCE-Ela structure.


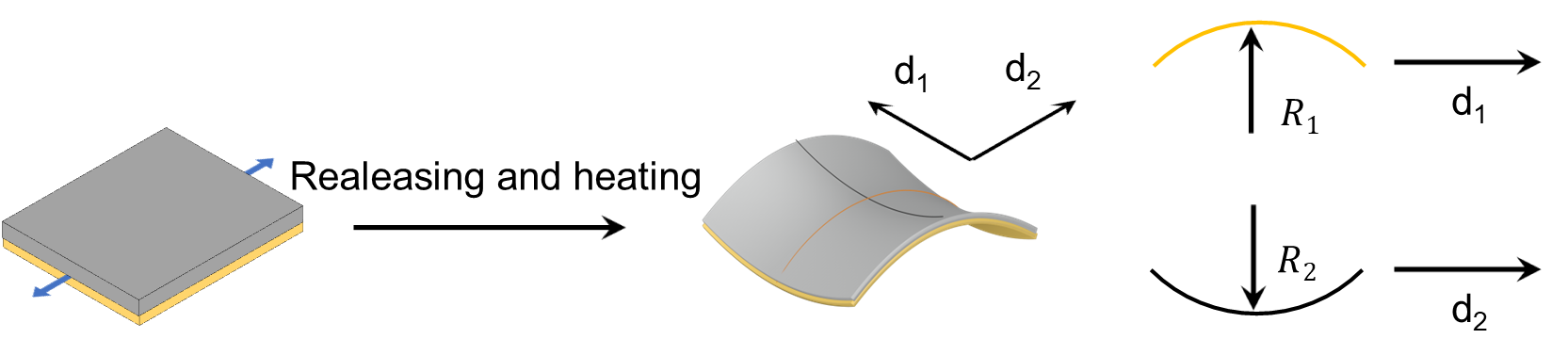


Figure S2. The two principal curvatures radius ($R_{1}$, $R_{2}$) are independently governed by strains along orthogonal principal directions ($d_{1}$, $d_{2}$).


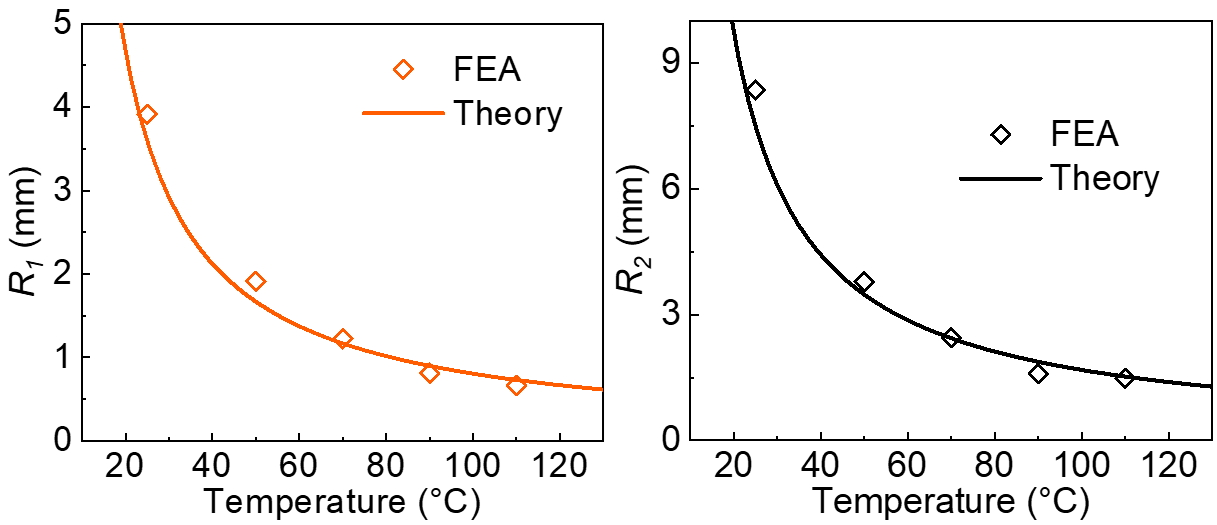


Figure S3. Comparison of FEA and theoretical results for the two principal curvatures radius ($R_{1}$, $R_{2}$).


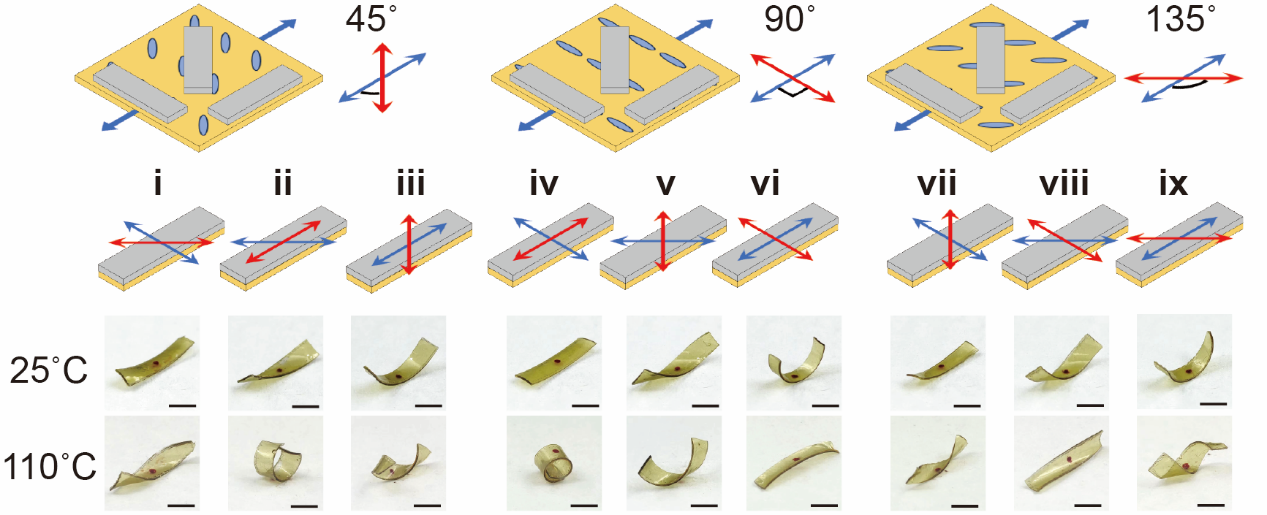


Figure S4. The 9 rectangular 2D precursors with varying pre-strain and thermal strain distributions, fabricated using three stretching methods and three printing positions, along with their assembled and driven shapes. All scale bar: 3 mm.


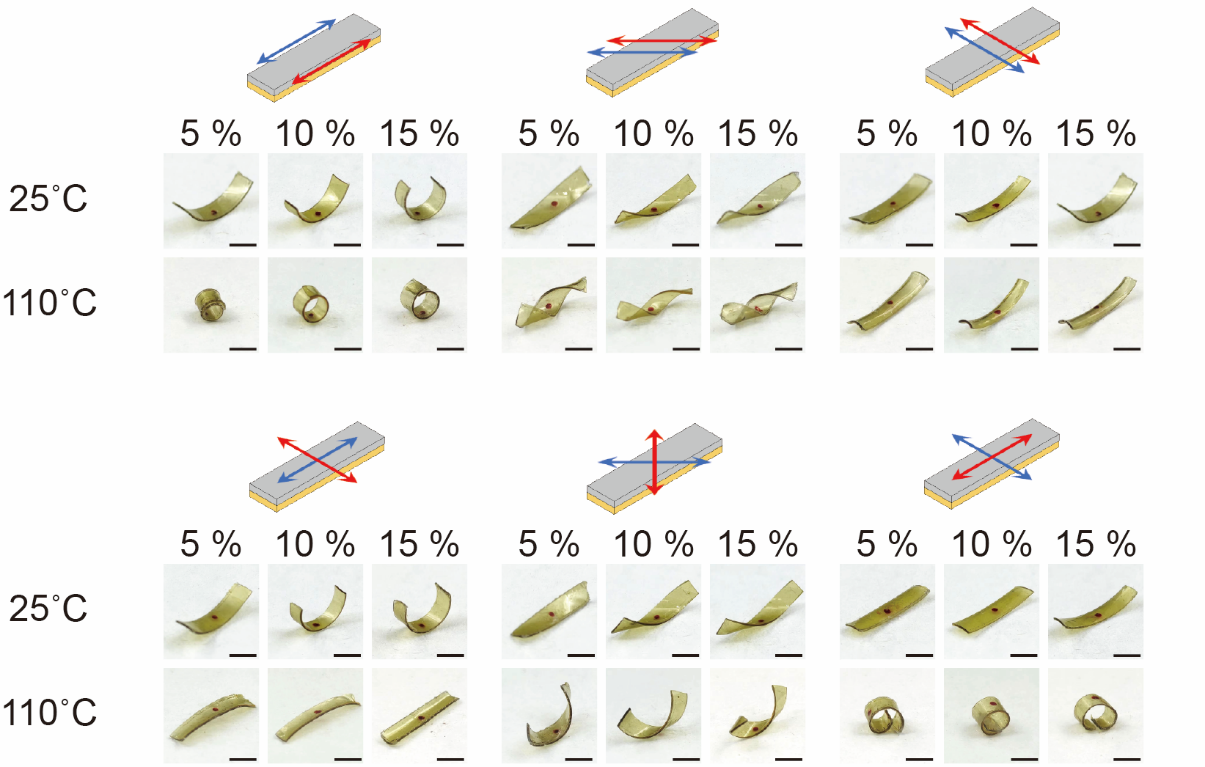


Figure S5. The assembled and driven shapes of rectangular 2D precursors with varying pre-strain direction (blue arrow) and molecular orientation (red arrow) under different prestrains (5%, 10% and 15%). All scale bar: 3 mm.


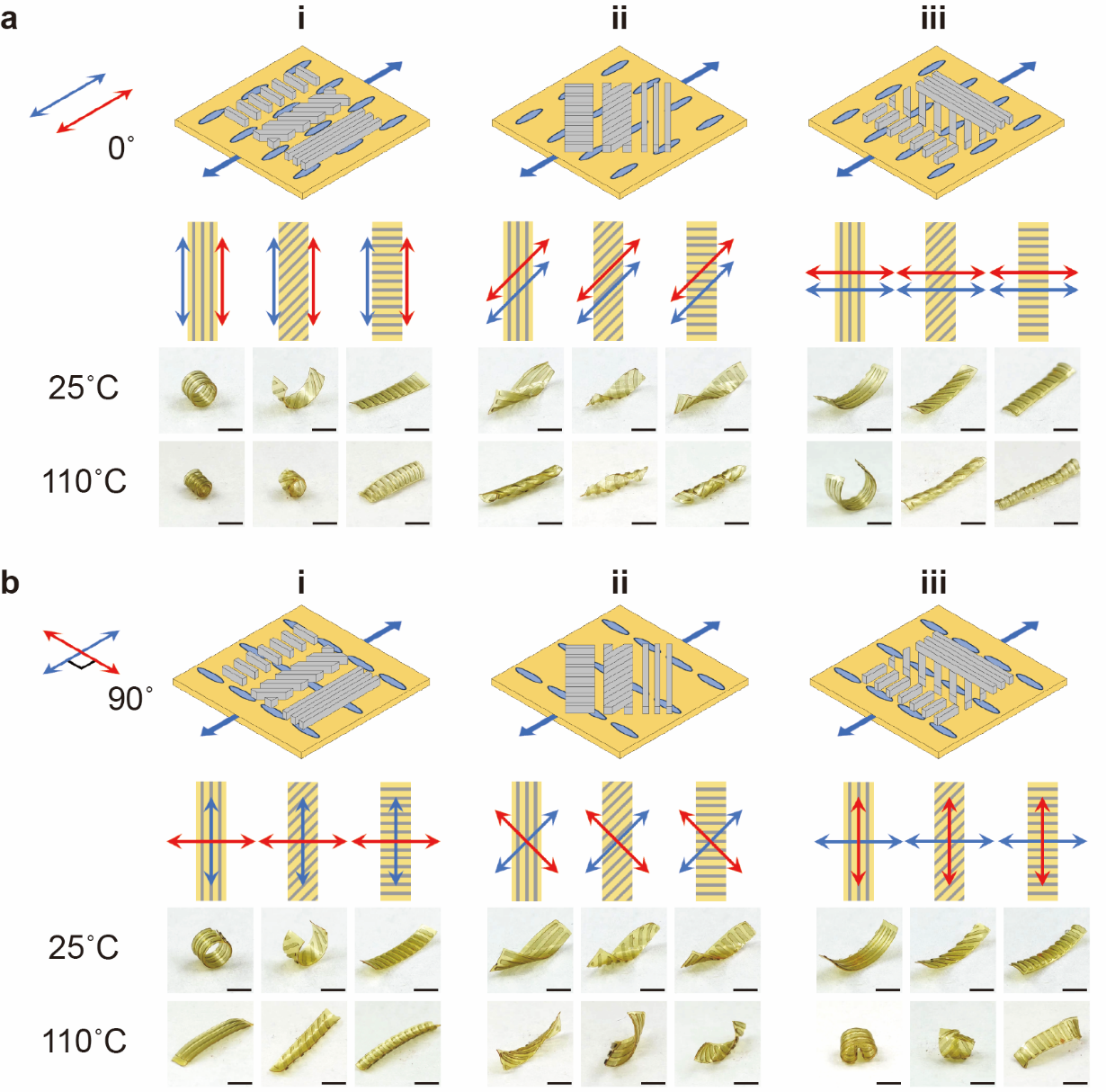


Figure S6. The assembly and actuation shape of the parallel-strip 2D precursors with different distributions of pre-strain and thermal strain. All scale bar: 3 mm.


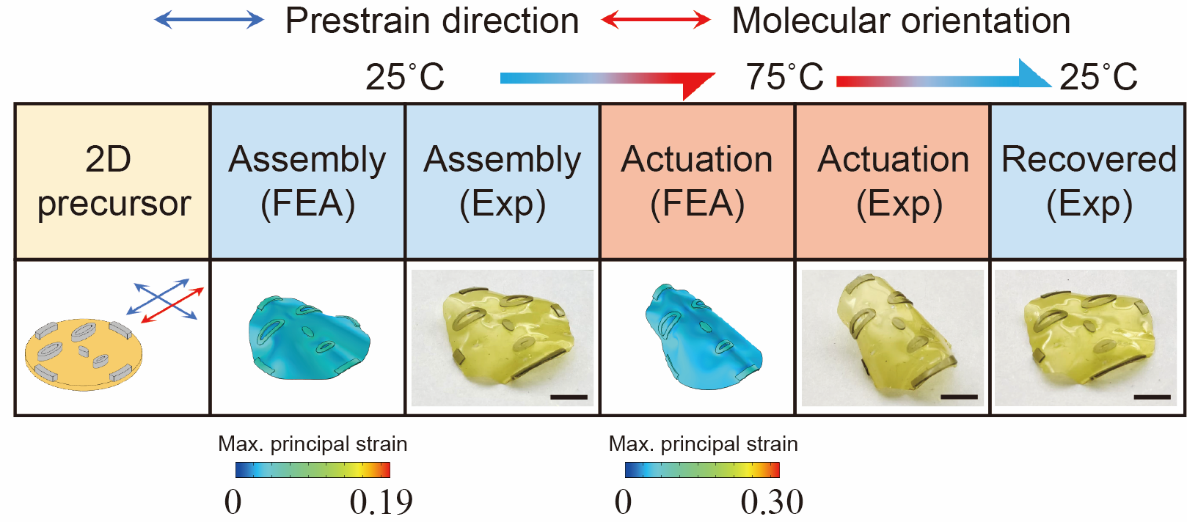


Figure S7. The Exp results and FEA predictions for the 3D LCE-Ela structures formed from the face-pattern 2D precursors, as well as its reversible 3D-to-3D shape transformations. All scale bar: 5 mm.


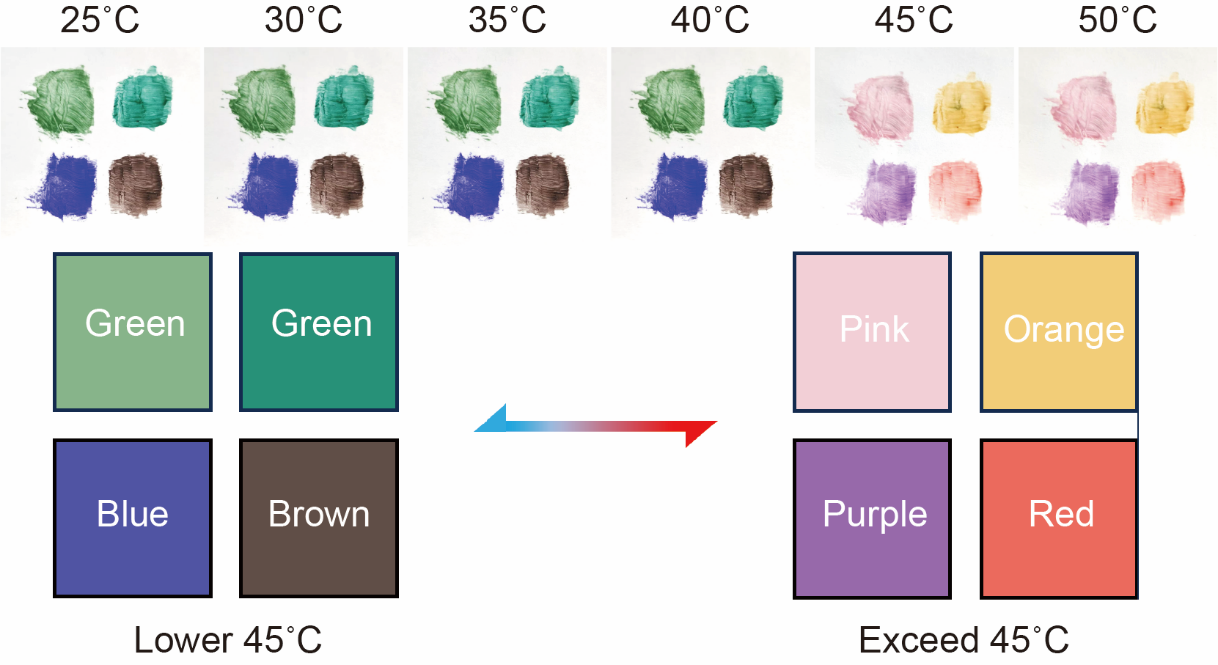


Figure S8. The colors of four different thermochromic inks at various temperatures. The color transition point for all inks is 45°C, and this color change is reversible.


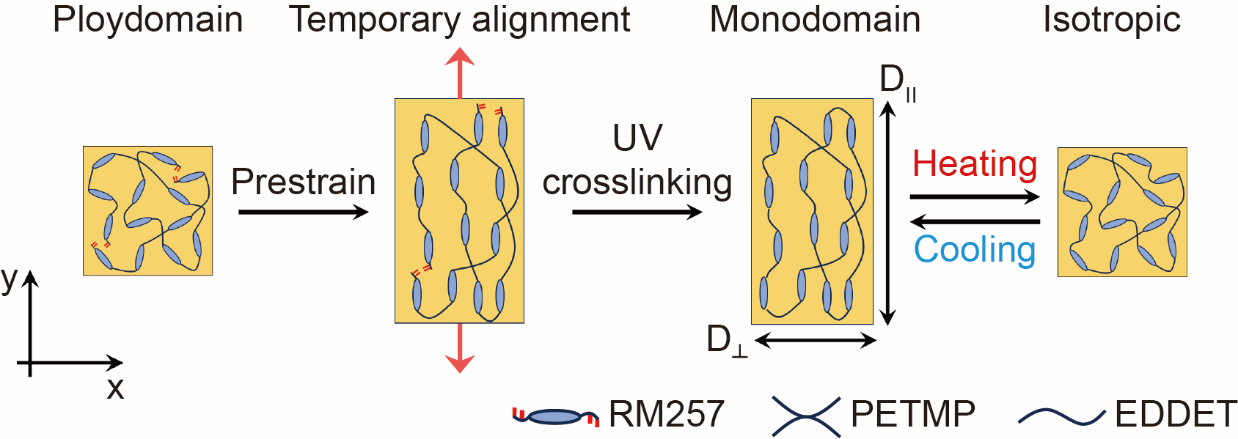


Figure S9. Processing LCE films followed by the two-stage thiol-acrylate Michael addition reaction methodology.


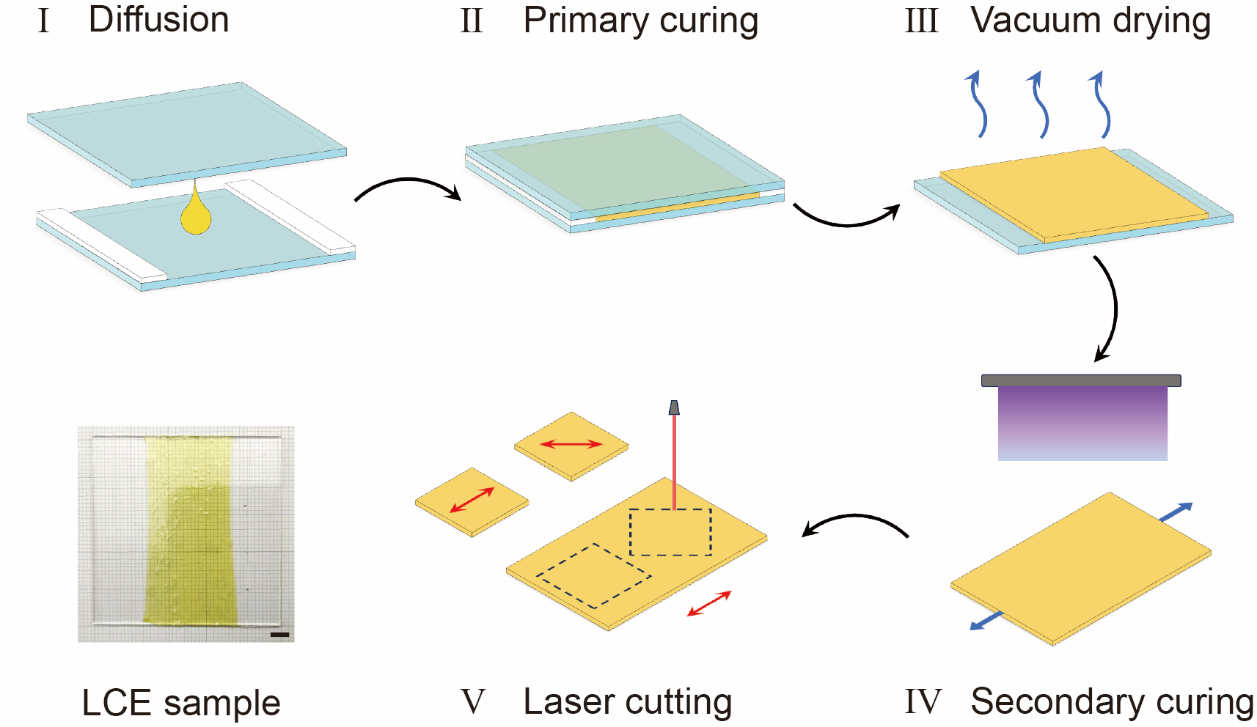


Figure S10. Schematic illustration depicting the fabrication process of LCE film (Ⅰ-Ⅴ) and the finally experiment sample of LCE films. All scale bar: 5 mm.


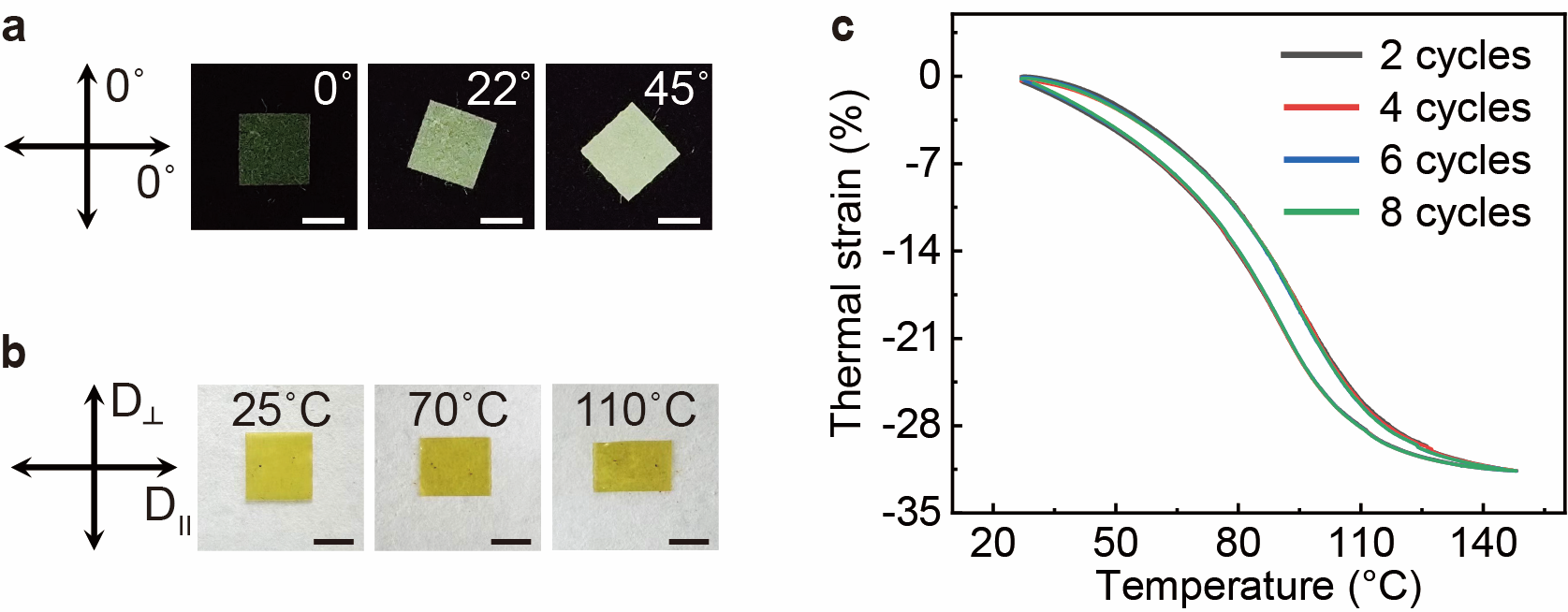


Figure S11. a) The birefringence character of the m-LCE film. The angular dependency of the transmitted light intensity indicates a well-aligned monodomain structure after the application of strain and second-stage polymerization. This suggests that the molecular arrangement is predominantly oriented along a specific direction. b) The square gradually transforms into a rectangle as the film is heated, exhibiting its anisotropic thermal deformation. c) The thermal strain-temperature curves of the LCE films along directions parallel ($D_{\parallel}$) under thermal cycling test. All scale bar: 3 mm.


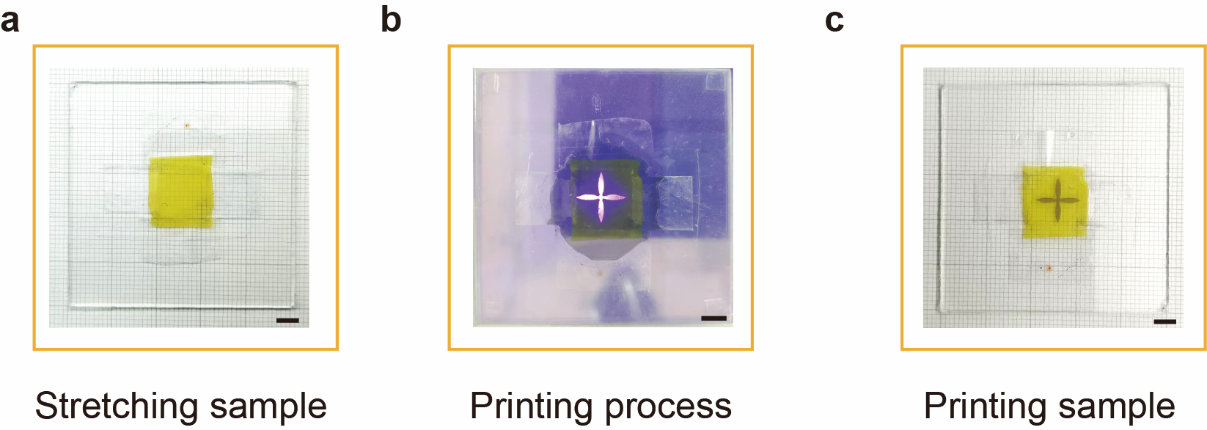


Figure S12. Fabrication of LCE-Ela 2D precursors. a) A sample of pre-stretched LCE fixed on a glass slide. b) Exposure process and (c) A sample of printing. All scale bar: 5 mm.


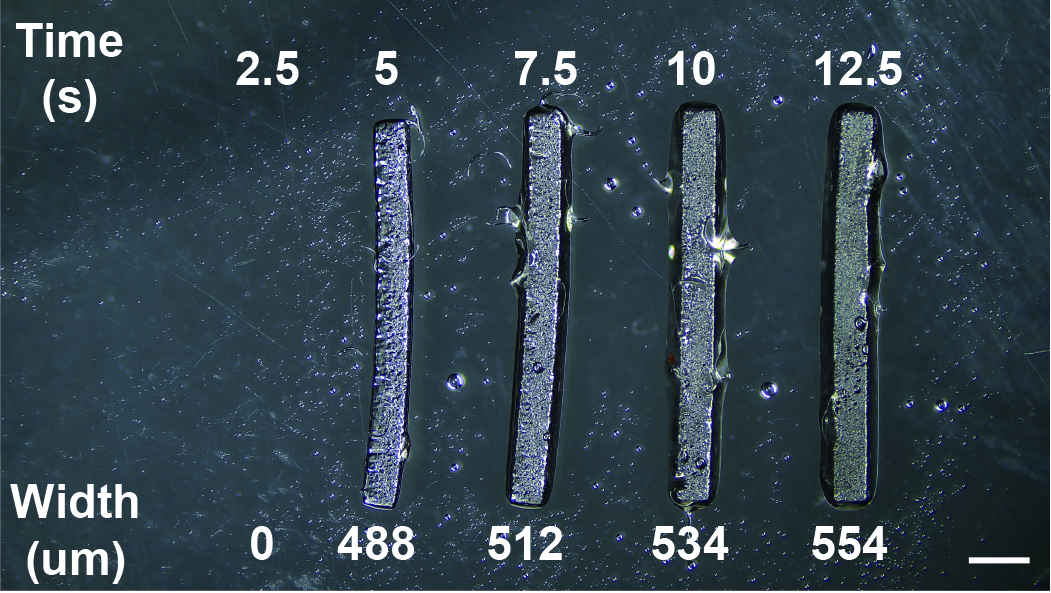


Figure S13. The influence of irradiation time on the degree of resin curing and width. All scale bar: 900 µm.


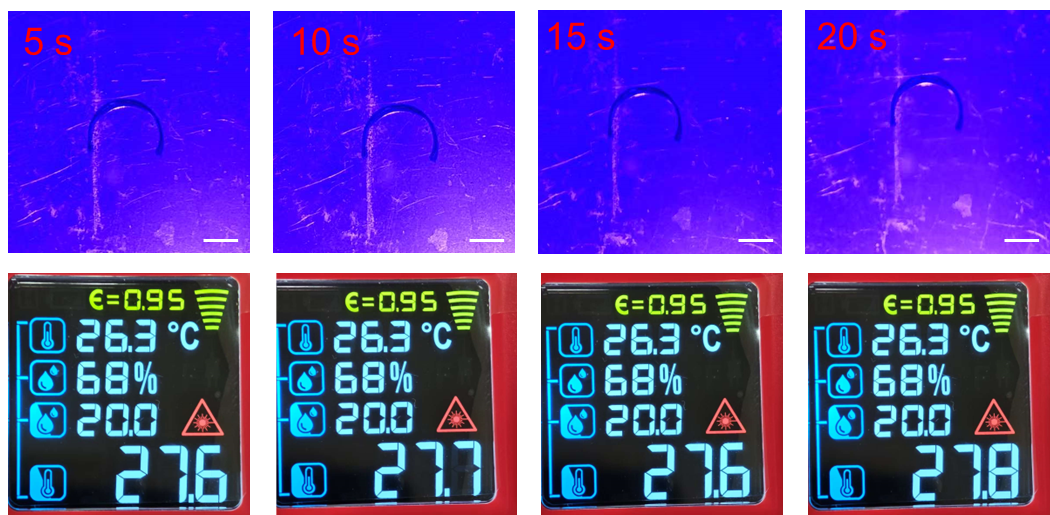


Figure S14. The influence of irradiation time on the temperature and deformation of specimen. All scale bar: 3 mm.


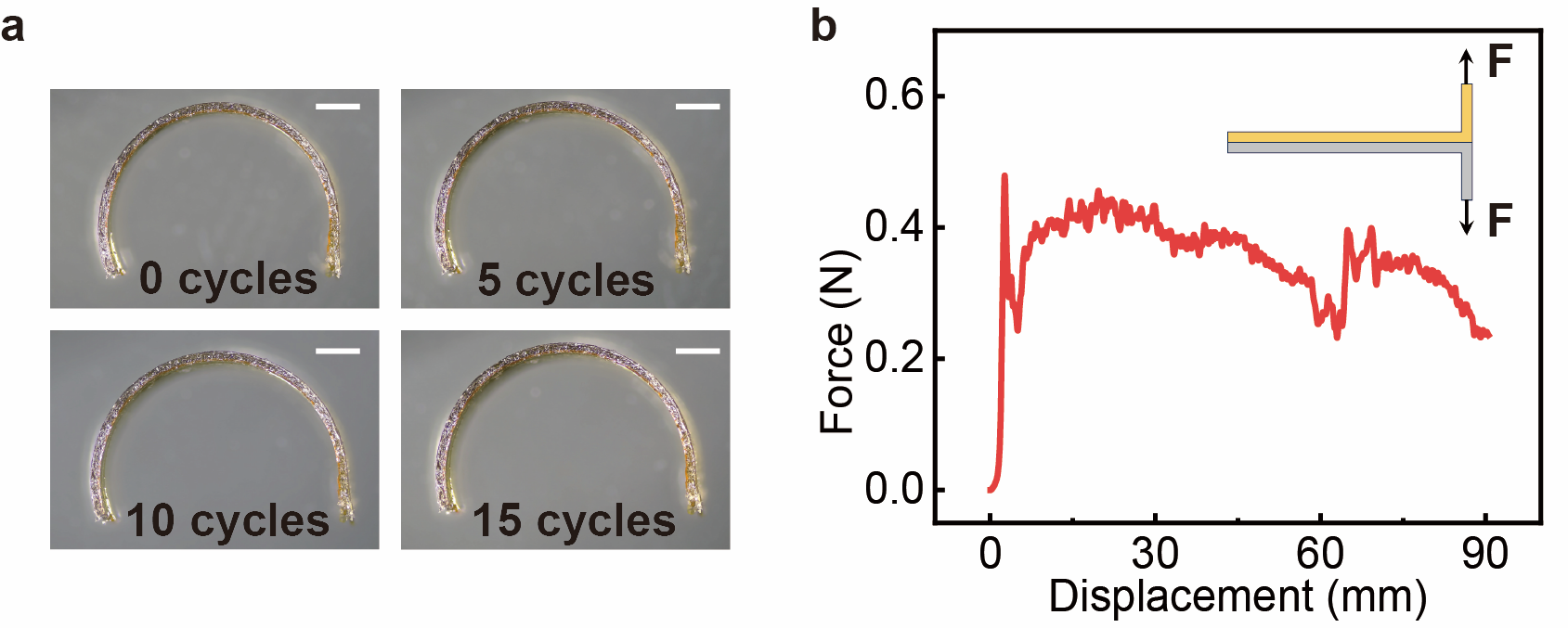


Figure S15. The bonding conditions of bilayer structure: (a) interfacial microstructure after multiple thermal cycles and (b) force-displacement curve of peel testing. All scale bar: 600 µm.


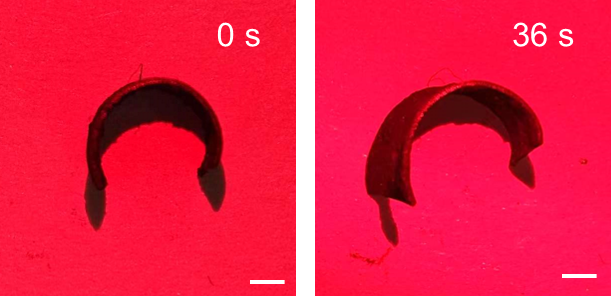


Figure S16. The infrared light-driven deformation of the LCE-Ela 3D structure. All scale bar: 2.5 mm.


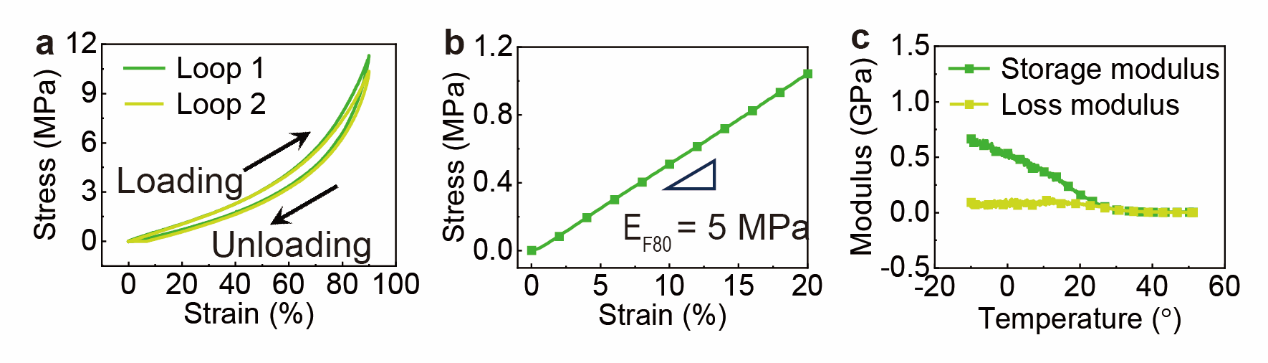


Figure S17. Material property of Ela. a) Loop test. b) Local stress-strain relation. c) Dynamic mechanical analysis.
